# A distinct *Fusobacterium nucleatum* clade dominates the colorectal cancer niche

**DOI:** 10.1038/s41586-024-07182-w

**Published:** 2024-03-20

**Authors:** Martha Zepeda-Rivera, Samuel S. Minot, Heather Bouzek, Hanrui Wu, Aitor Blanco-Míguez, Paolo Manghi, Dakota S. Jones, Kaitlyn D. LaCourse, Ying Wu, Elsa F. McMahon, Soon-Nang Park, Yun K. Lim, Andrew G. Kempchinsky, Amy D. Willis, Sean L. Cotton, Susan C. Yost, Ewa Sicinska, Joong-Ki Kook, Floyd E. Dewhirst, Nicola Segata, Susan Bullman, Christopher D. Johnston

**Affiliations:** 1 Vaccine and Infectious Disease Division, Fred Hutchinson Cancer Center, Seattle, WA USA; 2 Data Core, Shared Resources, Fred Hutchinson Cancer Center, Seattle, WA USA; 3 Human Biology Division, Fred Hutchinson Cancer Center, Seattle, WA USA; 4 Department of Computational, Cellular and Integrative Biology, University of Trento, Trento, Italy; 5 Korean Collection for Oral Microbiology and Department of Oral Biochemistry, School of Dentistry, Chosun University, Gwangju, Republic of Korea; 6 Department of Biostatistics, University of Washington, Seattle, WA USA; 7 Forsyth Institute, Cambridge, MA USA; 8 Department of Pathology, Dana-Farber Cancer Institute, Boston, MA USA; 9 Department of Oral Medicine, Infection and Immunity, Harvard School of Dental Medicine, Boston, MA USA

**Keywords:** Bacterial genomics, Colorectal cancer, Phylogeny, Pathogens

## Abstract

*Fusobacterium nucleatum* (*Fn*), a bacterium present in the human oral cavity and rarely found in the lower gastrointestinal tract of healthy individuals^1^, is enriched in human colorectal cancer (CRC) tumours^2–5^. High intratumoural *Fn* loads are associated with recurrence, metastases and poorer patient prognosis^5–8^. Here, to delineate *Fn* genetic factors facilitating tumour colonization, we generated closed genomes for 135 *Fn* strains; 80 oral strains from individuals without cancer and 55 unique cancer strains cultured from tumours from 51 patients with CRC. Pangenomic analyses identified 483 CRC-enriched genetic factors. Tumour-isolated strains predominantly belong to *Fn* subspecies *animalis* (*Fna*). However, genomic analyses reveal that *Fna*, considered a single subspecies, is instead composed of two distinct clades (*Fna* C1 and *Fna* C2). Of these, only *Fna* C2 dominates the CRC tumour niche. Inter-*Fna* analyses identified 195 *Fna* C2-associated genetic factors consistent with increased metabolic potential and colonization of the gastrointestinal tract. In support of this, *Fna* C2-treated mice had an increased number of intestinal adenomas and altered metabolites. Microbiome analysis of human tumour tissue from 116 patients with CRC demonstrated *Fna* C2 enrichment. Comparison of 62 paired specimens showed that only *Fna* C2 is tumour enriched compared to normal adjacent tissue. This was further supported by metagenomic analysis of stool samples from 627 patients with CRC and 619 healthy individuals. Collectively, our results identify the *Fna* clade bifurcation, show that specifically *Fna* C2 drives the reported *Fn* enrichment in human CRC and reveal the genetic underpinnings of pathoadaptation of *Fna* C2 to the CRC niche.

## Abstract (web summary)

A study reveals that *Fusobacterium nucleatum* subspecies *animalis* is bifurcated into two distinct clades, and shows that only one of these dominates the colorectal cancer niche, probably through increased colonization of the human gastrointestinal tract.

## Main

*Fn*, a member of the oral microbiota, has gained attention as an emerging cancer-associated bacterium. Worldwide, unbiased genomic analyses have revealed an enrichment of *Fn* in human CRC relative to non-cancerous colorectal tissues^9^. Previous work by our group and others demonstrated that patients with CRC tumours harbouring high levels of *Fn* have poorer survival^5^, that *Fusobacterium* colonizes regions of patient tumours with immune and epithelial cell functions supportive of cancer progression^10^, that *Fusobacterium* persists in metastatic disease^6^ and that microbiome modulation targeting *Fn* could change the course of this disease^6,11^. Moreover, exogenous *Fn* infection in animal and cellular models has supported a cancer-promoting role for this bacterium^6,9–14^. However, considerable strain-to-strain variation in *Fn* genotypic and phenotypic features has been described^12–16^. Such heterogeneity has raised challenges with reproducing *Fn* cancer-inducing phenotypes in animal and cellular models^16,17^ and it has been proposed that only a select group of *Fn* strains may possess carcinogenic capabilities^17^.

Here, leveraging a comprehensive collection of human CRC *Fn* strains and carrying out extensive comparative genomics, we reveal that a select clade within *Fn* subspecies predominates the CRC niche. In vitro and in vivo functional studies demonstrate that this clade is highly virulent in the context of CRC, and should be a primary focus in subsequent mechanistic studies on *Fn* pathogenicity in CRC and for the development of targeted inhibitors.

## Niche-enriched *Fn* genes and subspecies

**Fig. 1:**
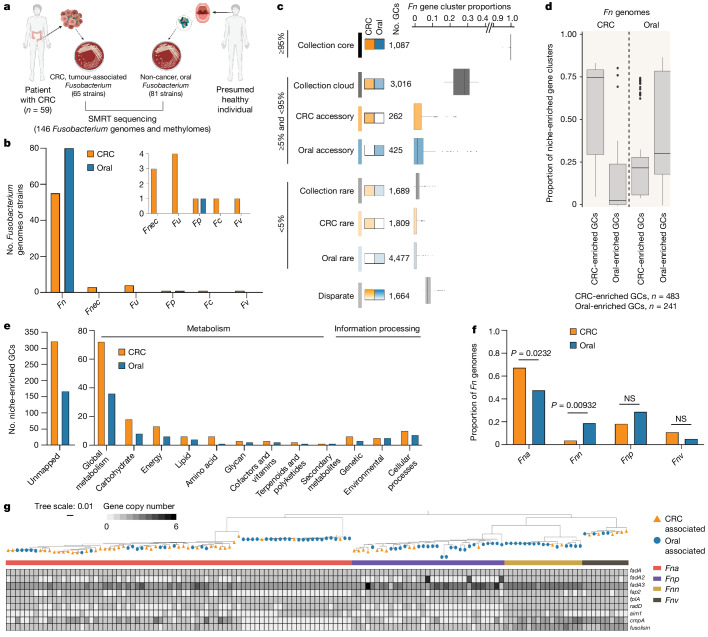
*Fn* niche features. **a**, A schematic of *Fusobacterium* strain collection (*n* = 146) and the sequencing strategy for unique strains. SMRT, single-molecule real-time sequencing. **b**, A column graph depicting the proportion of *Fusobacterium* genomes, subset by species, within the CRC (orange) and oral (blue) niches. The inset shows all non-*Fn* species of *Fusobacterium* (*Fnec*, *F.* *necrophorum*; *Fu*, *F.* *ulcerans*; *Fp*, *F.* *pseudoperiodonticum*; *Fc*, *F.* *canifelinum*; *Fv*, *F.* *varium*). **c**, The composition of the *Fn* pangenome subset by niche. Anvi’o^21^ gene cluster (GC) prevalence was used to define core (≥95%), accessory (≥5% and <95%) and rare (<5%) features conserved in both CRC-associated and oral-associated strains (collection core, ≥95% in all strains within the collection; collection cloud, ≥5% and <95% in all strains within the collection; collection rare, <5% in all strains within the collection). Disparate features are those that do not fall into any of the other noted bins. **d**, The proportion of niche-enriched gene clusters across CRC-associated and oral-associated *Fn* genomes. The plot box shows the 25th percentile, median and 75th percentile. The plot whiskers indicate the minima and maxima. **e**, KofamKOALA KEGG orthologue analysis^27^ of niche-enriched gene clusters. **f**, A column graph depicting the proportion of *Fn* genomes, grouped by subspecies, within the CRC and oral niche. Statistical analysis was carried out using a two-sample *z*-test, two-tailed. NS, not significant. **g**, Gene presence–absence heat map of canonical *Fn* virulence factors (*fadA* (refs. ^38,39,42^), *fap2* (ref. ^34^), *fplA* (ref. ^33^), *radD* (refs. ^36,62^), *aim1* (ref. ^35^), *cmpA* (ref. ^37^) and *fusolisin*^32^) across *Fn* subspecies, in which each column represents an individual genome (*Fna* *n* = 75, *Fnn* *n* = 17, *Fnp* *n* = 33, *Fnv* *n* = 10). The heat map is organized using an *rpoB* gene-based phylogenetic tree. For each genome, the tree end points indicate the niche origin (CRC (orange); oral (blue)) and the bar colour indicates the *Fn* subspecies (*Fna* (red); *Fnn* (gold); *Fnp* (purple); *Fnv* (brown)). The graphics in **a** were created using BioRender.com.

Classical microbiology culture approaches have re-emerged as valuable tools to functionally assess members of tissue-associated microbiomes. Here we carried out *Fusobacterium* targeted culture on 130 human CRC tumours from which 65 *Fusobacterium* CRC-associated strains were obtained from 59 unique patients. Given that *Fn* is predominantly an oral pathobiont, we included 81 *Fusobacterium* strains isolated from the oral cavity of individuals without cancer, as a control group. These oral strains were obtained from the American Type Culture Collection (ATCC) and the Korean Collection for Oral Microbiology (KCOM) repositories. Using PacBio long-read single-molecule real-time^18^ sequencing, we generated complete and closed genomes, with corresponding epigenetic methylomes, for 146 unique *Fusobacterium* strains (Fig. 1a and Supplementary Tables 1 and 2), 92% of which belonged to the species *Fn* (*n* = 135; *n* = 55 CRC associated and *n* = 80 oral associated; Fig. 1b). As *Fn* is the most frequently detected species in CRC tumours^2,3^, we therefore focused our analysis on a comparison of these 55 CRC-associated and 80 oral-associated *Fn* genomes.

**Extended Data Fig. 1:**
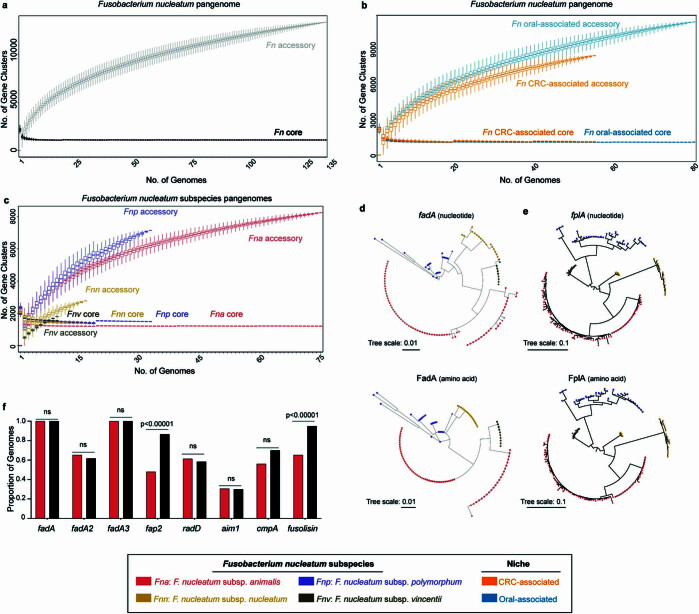
*Fn* genetic characterization by niche and subspecies. **a**, Size of the *Fn* pangenome split by the core genome (≥95%) (black) and accessory (<95%) genome (grey). *n* = 10,000 random subsamplings of 135 *Fn* genomes. Data is plotted as median ± s.d. **b**, Size of the *Fn* pangenome split by CRC-associated (orange) and oral-associated (blue) niche origin, with respective core and accessory genomes labeled. *n* = 10,000 random subsamplings of 55 *Fn* CRC-associated and 80 *Fn* oral-associated genomes. Data is plotted as median ± s.d. **c**, Size of the *Fn* pangenome split by *Fn* subspecies, *Fna* (red), *Fnn* (gold), *Fnp* (purple), *Fnv* (brown), with respective core and accessory genomes labeled. *n* = 10,000 random subsamplings of 75 *Fna*, 17 *Fnn*, 33 *Fnp*, and 10 *Fnv* genomes. Data is plotted as median ± s.d. **d**-**e**, Maximum-likelihood dendrograms of **d**, *fadA* and **e**, *fplA* nucleotide and amino acid sequences. For each genome, tree end points indicate *Fn* subspecies; *Fna* (red), *Fnn* (gold), *Fnp* (purple), *Fnv* (brown). **f**, Column graph depicts the proportion of *Fn* genomes containing canonical *Fn* virulence factors, subset by *Fna* (red), non-*Fna* (black) subspecies. Statistical analysis performed using two sample Z test, two-tailed. NS, not significant.

Given that *Fn* strains in human CRC tumours are predicted to originate from the human oral cavity^19,20^, but are rare members of the lower gastrointestinal (GI) tract microbiota of individuals without cancer^1^, we reasoned that CRC-associated *Fn* strains harbour an additional genetic repertoire to facilitate their colonization in human CRC tumours. To test this, we examined our 135 *Fn* genomes using the analysis and visualization platform for ‘omics data (Anvi’o) workflow for microbial pangenomics^21^. Pangenomic analysis identifies all genes present in a species (‘pangenome’) and discerns between gene content conserved among most members (‘core genome’) and gene content shared among subsets of members (‘accessory genomes’)^22,23^. We observed that accessory genome size increases as the number of sampled *Fn* genomes increases, supportive of previous proposals that *Fn* has an open pangenome^24^ (Extended Data Fig. 1a). To account for uneven sampling^25^ between CRC (*n* = 55) and oral (*n* = 80) genomes, we subset our analysis on the basis of niche and found that CRC-associated *Fn* strains have a smaller accessory genome compared to that of oral-associated strains (Fig. 1c, Extended Data Fig. 1b and Supplementary Table 1). Functional enrichment analysis^26^ identified 483 and 241 gene clusters significantly enriched (*q* < 0.05) in CRC and oral strains, respectively (Fig. 1d and Supplementary Table 3). Kyoto Encyclopedia of Genes and Genomes (KEGG) orthologue analysis^27^ of the 724 niche-enriched gene clusters revealed that mapped gene clusters (31.2%) were predominantly involved in putative metabolic functions and pathways (Fig. 1e and Supplementary Table 3).

Previously published studies using orthogonal approaches have observed a differential distribution of *Fn* subspecies in tumour tissue from patients with CRC and mucosal biopsy specimens from patients with inflammatory bowel disease^28,29^ (IBD). As there is sufficient genetic heterogeneity between the four *Fn* subspecies that reclassification into separate species has been proposed^30^, we increased the resolution of our analyses to the subspecies level (*Fna*, *Fn* subspecies *nucleatum* (*Fnn*), *Fn* subspecies *polymorphum* (*Fnp*) and *Fn* subspecies *vincentii* (*Fnv*); Methods). It is notable that studies seeking to delineate the contributions of *Fn* in CRC predominately use the model strains *Fnn* ATCC 25586 (ref. ^7^), an oral isolate, *Fnn* ATCC 23726 (refs. ^20,31^), a urogenital isolate, and *Fna* 7_1 (ref. ^29^), an isolate from a patient with IBD. Here, analysis of the proportion of *Fn* subspecies by niche found that of the four *Fn* subspecies, only *Fna* is significantly associated with the CRC niche (two-sample *z*-test, two-tailed, *P* = 0.0232; Fig. 1f), validating previous studies^28^, whereas *Fnn* is significantly enriched in the oral niche (two-sample *z*-test, two-tailed, *P* = 0.00932; Fig. 1f). Thus, we reasoned that the repertoire of genetic factors associated with colonization or virulence in the CRC niche would not be fully represented in *Fnn* model strains and indeed show that *Fnn* ATCC 25586 contains only 17.60% of CRC-enriched gene clusters (Supplementary Table 3). This supports the use of *Fna* strains such as *Fna* 7_1, which has been shown to induce colonic tumours in mouse models^3^, for mechanistic studies. Comparison of *Fn* subspecies pangenomes showed that *Fna* has the smallest core genome compared to those of other subspecies, suggestive of further unresolved *Fna* genetic heterogeneity (Extended Data Fig. 1c).

As *Fna* is enriched in the CRC tumour niche, we tested whether *Fn* virulence factors previously described as important for host colonization are more prevalent in *Fna* than in other *Fn* subspecies. *Fn* type Va autotransporter virulence factors include fusolisin^32^, a serine protease that damages host tissue and inactivates immune effectors, FplA (ref. ^33^), a phospholipase autotransporter that binds to host phosphoinosite-signalling lipids, and the Fap2 (ref. ^34^), Aim1 (ref. ^35^), RadD (ref. ^36^) and CmpA (ref. ^37^) adhesins that mediate *Fn* interactions with either host cells or other bacterial species. As the role of *fplA*, *aim1*, *radD* and *cmpA* in CRC remains unclear, to ensure a comprehensive analysis, we queried the presence of these virulence factors across our *Fn* genome collection. An additional adhesin, FadA (refs. ^38,39^), mediates *Fn* attachment to and invasion of host epithelial cells^40,41^, with two additional FadA homologues recently identified^42^. Previously analysed *fadA* distribution in a limited number of *Fusobacterium* genomes has suggested *fadA* absence from passively invading species and increased incidence in highly invasive species^13^, although this distribution does not always coincide with in vivo invasion assays^13,38,42,43^. We found that although *fplA* and *fadA* are well conserved in *Fn*, their nucleotide and amino acid sequences segregate by *Fn* subspecies, perhaps indicative of variable interactions with host ligands (Extended Data Fig. 1d,e). Our results show that none of these canonical virulence factors is significantly associated with *Fna* compared to other *Fn* subspecies, suggesting that additional unknown genetic factors facilitate the enrichment of *Fna* in CRC (Extended Data Fig. 1f). However, we observed that a subset of *Fna* strains lacked *fap2*, *cmpA* and *fusolisin* and, by *rpoB* gene analysis, these *Fna* strains seemed to form a distinct *Fna* clade (Fig. 1g).

## An *Fna* clade enriched in the CRC niche

**Fig. 2:**
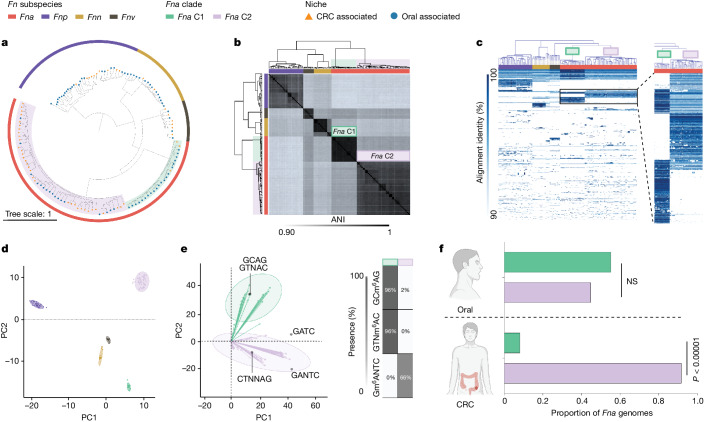
Genetic and epigenetic characteristics of *Fna* clades. **a**, A kSNP^45^ maximum-likelihood whole-genome phylogenetic tree. For each *Fn* genome (*n* = 135), the tree end points indicate the niche origin (CRC (orange); oral (blue)) and the bar colour indicates the *Fn* subspecies (*Fna* (red); *Fnn* (gold); *Fnp* (purple); *Fnv* (brown)). Within *Fna*, the background colour indicates the *Fna* clade (*Fna* C1 (green); *Fna* C2 (lavender)). **b**, A clustered dendrogram of the ANI matrix. The bar colour indicates the *Fn* subspecies (*Fna* (red); *Fnn* (gold); *Fnp* (purple); *Fnv* (brown)). The *Fna* clades are highlighted with green and lavender boxes. ANI values are reported in Supplementary Tables 4 and 5. **c**, A GiG-map visualization of the protein-coding gene content across *Fn* genomes. The top bar colour indicates the *Fn* subspecies (*Fna* (red); *Fnn* (gold); *Fnp* (purple); *Fnv* (brown)) and the box colour indicates the *Fna* clade (*Fna* C1 (green); *Fna* C2 (lavender)). The inset on the right highlights groups of protein-coding genes that are distinct between *Fna* C1 and *Fna* C2. An interactive GiG-map dataset is available at https://fredhutch.github.io/fusopangea/. **d**, PCA of Anvi’o gene clusters by presence and absence in each genome. The colours indicate the *Fn* subspecies and *Fna* clades (*Fnn* (gold); *Fnp* (purple); *Fnv* (brown); *Fna* C1 (green); *Fna* C2 (lavender)). The ellipses are drawn to 95% confidence. **e**, Left: PCA of *Fna* genome-wide methyl-modified nucleotide sequences. The ellipses are drawn to 95% confidence. The overlay of the PCA biplot shows the top five nucleotide motifs that are driving the *Fna* clade bifurcation. Right: a table indicating the distribution of each motif across the *Fna* clades. The colour indicates the *Fna* clade (*Fna* C1 (green); *Fna* C2 (lavender)). **f**, A column graph depicting the proportion of *Fna* CRC-associated and *Fna* oral-associated genomes, subset by *Fna* clade (*Fna* C1 (green); *Fna* C2 (lavender)). The statistical analysis was carried out using a two-sample *z*-test, two-tailed. NS, not significant. The graphics in **f** were created using BioRender.com.

**Extended Data Fig. 2:**
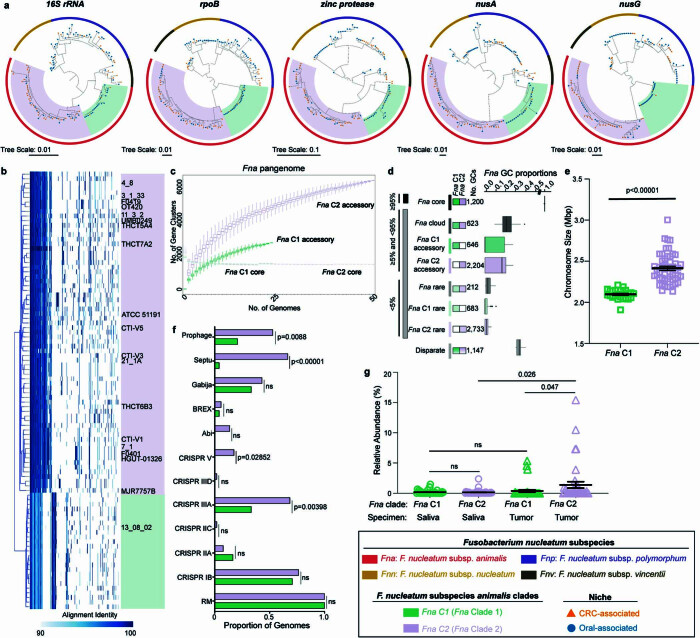
Phylogenetics and genomics of *Fna* clades. **a**, Maximum-likelihood phylogenetic trees of *Fn* single marker genes, *16* *S rRNA*, *rpoB*, *zinc protease*, *nusA* and *nusG*. For each genome (n = 135), tree end points indicate niche origin, CRC (orange) or oral (blue), bar color indicates *Fn* subspecies (*Fna* (red), *Fnn* (gold), *Fnp* (purple), *Fnv* (brown)), and background color indicates *Fna* clades (*Fna* C1 (green) and *Fna* C2 (lavender)). **b**, Genes-in-genomes map (GiG-map) visualization of protein coding gene content across *Fna* genomes. Boxes highlight *Fna* clades, *Fna* C1 (green) and *Fna* C2 (lavender). Previously published NCBI genomes are labeled by strain name. **c**, Size of the *Fna* pangenome split by *Fna* clade, *Fna* C1 (green) and *Fna* C2 (lavender), with respective core and accessory genomes labeled. *n* = 10,000 random subsamplings of 24 *Fna* C1 and 51 *Fna* C2 genomes. Data is plotted as median ± s.d. **d**, Composition of *Fna* pangenome subset by clade. Anvi’o gene cluster (GC) prevalence was used to define core (≥95%), accessory (≥5% and <95%), and rare (<5%) features conserved in both *Fna* C1 and *Fna* C2 strains (“*Fna* core” (≥95% in all *Fna* strains), “*Fna* cloud” ( ≥ 5% and <95% in all *Fna* strains), “*Fna* rare” (<5% in all strains) or unique for strains from each clade. Disparate features are those that do not fall into any of the other noted bins. Plot box shows 25^th^ percentile, median, and 75^th^ percentile. Plot whiskers indicate minima and maxima. **e**, Column graph indicates chromosome sizes in *Fna* C1 (n = 24) and *Fna* C2 (n = 51). Data is plotted as mean ± s.e.m. Statistical analysis performed using Welch’s T-test, two-tailed. **f**, Column graph depicts the proportion of *Fna* genomes containing innate bacterial genetic defense systems, subset by *Fna* clades, *Fna* C1 (green) and *Fna* C2 (lavender). Statistical analysis performed using two sample Z test, two-tailed. NS, not significant. **g**, Graph shows the percent relative abundance of *Fna* C1 (green) and *Fna* C2 (lavender) in paired saliva (circle) or tumor biopsy (triangle) samples from 39 patients with colorectal adenocarcinomas^47^. Data is plotted as mean ± s.e.m. Statistical analysis performed using Welch’s T-test, paired. NS, not significant.

To further examine the observation that *Fna* strains form two distinct clades, we compared phylogenetic trees of housekeeping genes previously used for *Fn* subspecies typing^28,44^. Analysis of these single-marker genes supported the observation that two distinct *Fna* clades exist, which we call *Fna* clade 1 (*Fna* C1) and *Fna* clade 2 (*Fna* C2; Extended Data Fig. 2a). Beyond these single-marker genes, genome-wide differences between *Fna* clades are supported by a kSNP^45^ reference-free whole-genome phylogeny (Fig. 2a). To quantify the relatedness of *Fna* C1 to *Fna* C2, we compared the average nucleotide identity (ANI), a well-established index that measures the percentage of similarity between genomes, with an established 95% species threshold^46^. Between *Fna* clades the ANI ranged from 91.61% to 93.11%, comparable to the ANI between other *Fn* subspecies (Fig. 2b and Supplementary Tables 4 and 5). Further, we visualized the patterns of protein-coding genes present across *Fna* genomes using the Genes in Genomes-Map (GiG-map) tool and found that *Fna* C1 and *Fna* C2 have distinct protein-coding gene content (Fig. 2c). This was further supported by principal component analysis (PCA) of Anvi’o gene cluster presence–absence (Fig. 2d). Notably, the frequently used *Fna* 7_1 strain groups with *Fna* C2 (Extended Data Fig. 2b).

Therefore, we reassessed the genetic, epigenetic and ecological properties of *Fna* as two genetically distinct clades. Comparison of the *Fna* clade pangenomes showed that *Fna* C1 and *Fna* C2 had similar core genome sizes, although *Fna* C2 had a larger accessory genome (Extended Data Fig. 2c,d), suggesting that *Fna* C2 strains harbour additional genetic factors that may be beneficial during colonization of CRC tumours. Consistent with this, comparisons of individual genome size and content indicated that *Fna* C2 strains have significantly larger chromosome sizes (Welch’s *t*-test, two-tailed, *P* < 0.00001; Extended Data Fig. 2e), more extrachromosomal plasmids (Supplementary Tables 1 and 6) and a greater number of innate genetic defences and mobile genetic elements (Extended Data Fig. 2f) compared to *Fna* C1 strains. PCA analysis of *Fna* methylomes indicated that *Fna* C1 and *Fna* C2 are also epigenetically distinct (Fig. 2e). The methyl-modified DNA motifs most influencing this epigenetic bifurcation are GTN^m^^6^AC (100% *Fna* C1, 0% *Fna* C2), GC^m^^6^AG (100% *Fna* C1, 0% *Fna* C2) and G^m^^6^ANTC (0% *Fna* C1, 63% *Fna* C2; Fig. 2e and Supplementary Table 7). Although both *Fna* clades are present in the oral cavity with non-significant differences, only *Fna* C2 is significantly associated with the CRC niche (two-sample *z*-test, two-tailed, *P* < 0.00001; Fig. 2f). We further tested this observation on publicly available 16S rRNA gene sequencing data from paired tumour tissue and saliva samples from patients with CRC^47^. To gain resolution to the *Fna* clade level, we identified *Fna* clade-specific amplicon sequence variants^48^ (Methods and Supplementary Table 8). Supportive of our observations, our data show that *Fna* C2 is significantly enriched in tumour samples compared to *Fna* C1 (*t*-test, paired, *P* = 0.047; Extended Data Fig. 2g). However, there was no statistically significant difference between *Fna* clades in paired oral samples, indicating that although both *Fna* clades are present in the oral cavity of patients with CRC, only *Fna* C2 is enriched in the tumour niche (Extended Data Fig. 2g).

## Human lower GI tract *Fna* C2 enrichment

**Fig. 3:**
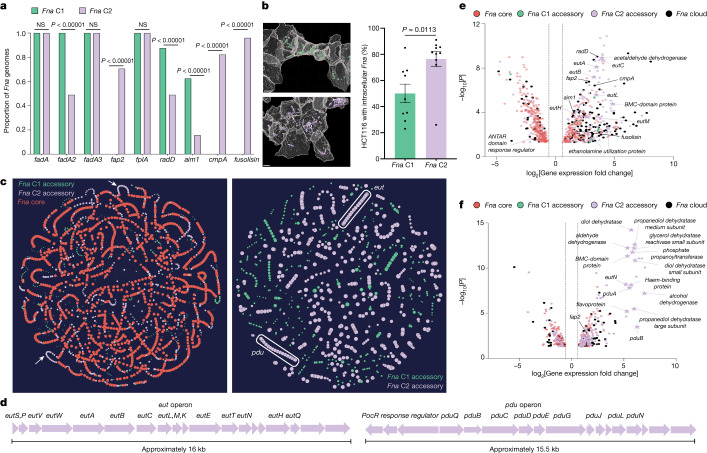
Inter *Fna*-clade comparative analyses. **a**, A gene presence versus absence column graph depicting the proportion of *Fna* genomes containing canonical *Fn* virulence factors, subset by clade (*Fna* C1 (green); *Fna* C2 (lavender)). The statistical analysis was carried out using a two-sample *z*-test, two-tailed. **b**, Left: computational confocal analysis of colon cancer epithelial cells (HCT116; grey) co-incubated with representative *Fna* C1 (green) or *Fna* C2 (lavender) strains. Scale bars, 4 μm. Right: a bar plot demonstrating the percentage of HCT116 cells with intracellular *Fna*; *n* = 3 biological replicates with 3 analysed *z*-stacks. Data are plotted as mean ± s.e.m. The statistical analysis was carried out using Welch’s *t*-test, two-tailed. **c**, A PPanGGOLiN^51^ map of the *Fna* pangenome. Each node represents a gene group, syntenic nodes represent neighbouring genes, the size indicates relative presence across *Fna* genomes, and the colour depicts pangenome partition (*Fna* core (red); *Fna* C1 accessory genome (green); *Fna* C2 accessory genome (lavender)). The white arrows (left panel) and oblongs (right panel) indicate *Fna* C2-associated putative *eut* and *pdu* operons. **d**, Schematics of these *Fna* C2 operons. An interactive PPanGGOLiN map is available at https://fredhutch.github.io/fusopangea/. **e**,**f**, Differentially expressed genes (log_2_[fold change] ≥ 0.58 and ≤−0.58 with −log_10_[*P* value] ≥ 1.30) in a representative *Fna* C2 strain, SB010, exposed to EA (**e**) or 1,2-PD (**f**) compared to unexposed SB010 control. To highlight SB010-unique content, genes also differentially expressed under the same exposure conditions in a representative *Fna* C1 strain, KCOM 3764, have been removed (Extended Data Fig. 4b,c). The vertical dotted lines indicate the threshold of significant gene expression, defined as log_2_[fold change] ≥ 0.58 and ≤−0.58. The statistical analysis was carried out using glmQLFTest, two-sided. The data point colours indicate *Fna* core (red), *Fna* C1 accessory (green), *Fna* C2 accessory (lavender) or *Fna* cloud (black; present in ≥5% and <95% in all *Fna* strains) genes. The stars indicate *eut* and *pdu* operon genes.

**Extended Data Fig. 3:**
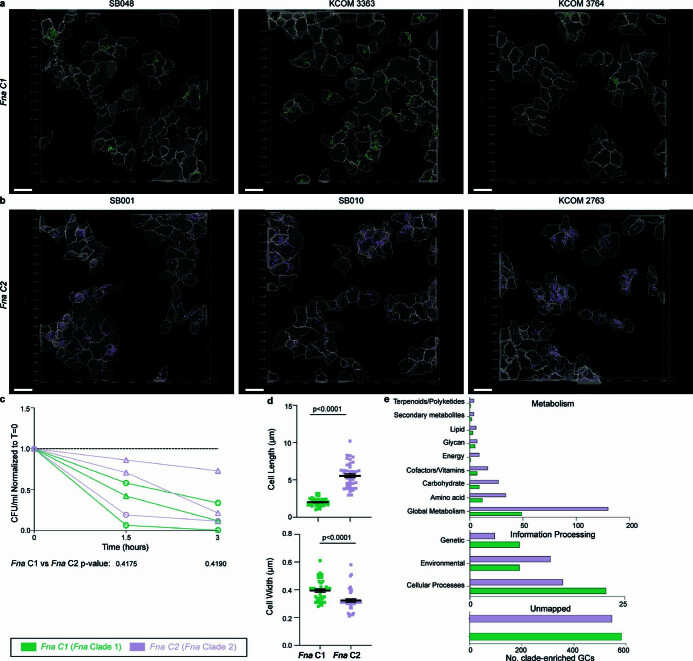
Morphological and genomic differences between *Fna* clades. Representative *Fna* C1 and *Fna* C2 strains co-cultured with human colon cancer cells (HCT116). **a**-**b**, Computational analysis of confocal imaging. Independent masks for cancer epithelial cells (grey), and intracellular bacterial cells (*Fna* C1 green; *Fna* C2 lavender) were generated. Masks were used to calculate the percent of HCT116 cells with intracellular *Fna* (Fig. 3b) (see Methods). Scale bar is 20 μm. **c**, Bacterial aerotolerance was assessed through serial dilution plating at start, mid-point, and endpoint of co-culture. Graph shows resulting bacterial colony forming units per millilter, standardized to start point for each strain. Dashed line indicates normalization equal to one. Statistical analysis performed using a Welch’s T-test, two-tailed. **d**, Bar plots indicate *Fna* cell length and cell width as measured from confocal microscopy images, subset by *Fna* clades, *Fna* C1 (green) or *Fna* C2 (lavender); n = 45 cells per *Fna* clade. Data is plotted as mean ± s.e.m. Statistical analysis performed using Welch’s T-test, two-tailed. **e**, KofamKOALA KEGG^27^ ortholog mapping of *Fna* clade-enriched gene clusters.

Pangenome analysis revealed that *Fna* is composed of two distinct clades but only *Fna* C2 is enriched in the CRC niche. Low levels of *Fna* C1 in this niche could be due to poor tumour colonization potential, an inherent lack of virulence or tumour-supportive factors that are possessed by *Fna* C2, or *Fna* C1 being unable to evade immune clearance. To interrogate these possibilities and reveal *Fna* clade-specific genetic factors, we applied a comprehensive inter-*Fna* clade comparative analysis across all 75 *Fna* genomes (24 *Fna* C1, 51 *Fna* C2). Canonical *Fn* virulence factors including the adhesins encoded by *radD*, *aim1* and *fadA2* were significantly enriched in *Fna* C1 compared to *Fna* C2 (two-sample *z*-test, two-tailed, *P* < 0.00001; Figs. 1g and 3a) suggesting that their role may be particularly important in the oral cavity. Conversely, as noted, *fap2*, *cmpA* and *fusolisin* are absent from *Fna* C1 and significantly associated with *Fna* C2 (two-sample *z*-test, two-tailed, *P* < 0.00001; Figs. 1g and 3a). Given the reported epithelial^31^ and immune cell^49^ interactions of Fap2 in CRC, its association with *Fna* C2 supports its increased adherence and invasion potential in this niche. Co-culture of *Fna* strains from each clade with a human colon cancer cell line (HCT116) demonstrate that *Fna* C2 has a significantly higher level of cancer epithelial cell invasion compared to *Fna* C1 strains (Fig. 3b and Extended Data Fig. 3a,b; Welch’s *t*-test, two-tailed, *P* = 0.0113), indicative of differential invasion potential and/or aerotolerance of individual strains (Extended Data Fig. 3c). Further, the *Fna* clades are morphologically distinct, with *Fna* C2 cells being significantly longer (*Fna* C1: 2.01 μm average, *Fna* C2: 5.26 μm average) and thinner (*Fna* C1: 0.39 μm average, *Fna* C2: 0.33 μm average) than *Fna* C1 cells (Extended Data Fig. 3d; Welch’s *t*-test, two-tailed, *P* < 0.00001 length, *P* < 0.00001 width). As bacterial morphology can affect colonization of host niches and susceptibility to host defences^50^, physical differences between *Fna* clade cells are noteworthy.

We reasoned that delineation of *Fna* clade-unique genome content could reveal hitherto unknown genetic factors enabling *Fna* C2 transit to and survival within the human colonic niche. Predominant genetic differences between *Fna* clades are consistent with *Fna* C2 having increased nutrient scavenging mechanisms and enhanced metabolic potential (Extended Data Fig. 3e and Supplementary Table 9). As functionally related bacterial genes often form co-regulated units (operons), we implemented the Partitioned PanGenome Graph of Linked Neighbors (PPanGGOLiN)^51^ tool to assess whether *Fna* clade-unique genetic factors formed putative operons (Fig. 3c). Consistent with Anvi’o analysis, the PPanGGOLiN analysis showed that *Fna* C2 syntenic blocks were predominantly associated with metabolic mechanisms (Supplementary Table 10). Thus, the pathoadaptation of *Fna* C2 to the CRC niche is multifactorial, and in addition to canonical *Fn* virulence factors is potentially facilitated by enhanced metabolic capabilities.

**Extended Data Fig. 4:**
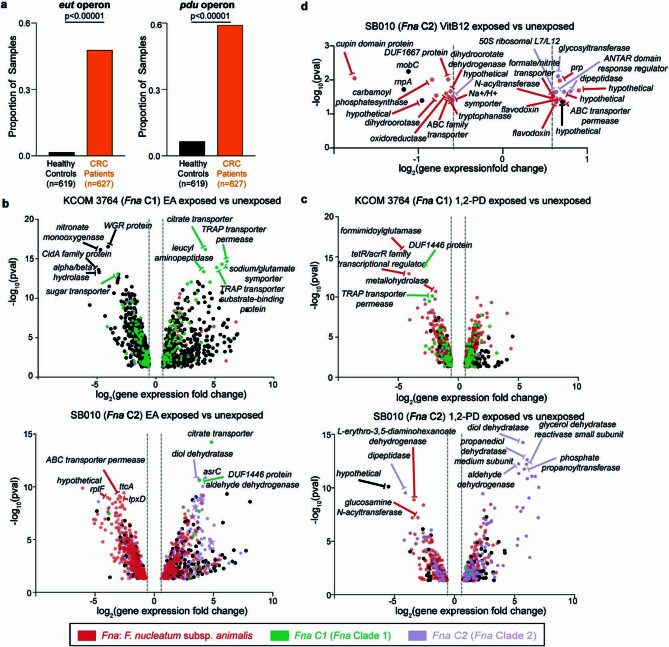
*Fna* clade transcriptomic responses to intestinal metabolites. **a**, Bar plots demonstrate the proportion of stool metagenomic samples from patients with CRC or healthy controls in which putative *eut* and *pdu* operons were detected. Statistical analysis performed using two sample Z test, two-tailed. Cohort sample sizes are indicated at the bottom of each panel. **b-c**, Differentially expressed genes (with log2-transformed fold change ≥ 0.58 and ≤ −0.58 with -log10(p-value) ≥ 1.30) of a representative *Fna* C1 strain (KCOM 3764) and a representative *Fna* C2 strain (SB010) under (**b**) ethanolamine (EA) or (**c**) 1,2-propanediol (1,2-PD) exposure as compared to their respective unexposed controls. Top five significant (-log10(p-value) ≥ 1.30) upregulated and downregulated genes are labeled. **d**, Differentally expressed genes (with log2-transformed fold change ≥ 0.58 and ≤ −0.58 with -log10(p-value) ≥ 1.30) in SB010 under Vitamin B12 exposure alone as compared to unexposed control. All differentially expressed genes labeled. For **b**-**d**, vertical dotted lines indicate the threshold of significant gene expression, defined as log2-transformed fold change ≥ 0.58 and ≤ −0.58. Statistical analysis performed using glmQLFTest, 2-sided. Data point colors indicate whether a gene is categorized as part of the *Fna* core genome (red), *Fna* C1-associated accessory genome (green), or *Fna* C2-associated accessory genome (lavender) by PPanGGOLiN.

To validate our inter-*Fna* clade pangenomic approach, we focused on two *Fna* C2-associated putative operons consistent with ethanolamine (EA) metabolism (*eut*) and 1,2-propanediol (1,2-PD) metabolism (*pdu*; Fig. 3d). These operons contribute about 20% of *Fna* C2-unique gene content (Supplementary Table 10). Enteric pathogens not only gain a competitive advantage through direct metabolism of EA and 1,2-PD, but also exploit their intestinal specificity. Sensing through *eut* and *pdu* activates global regulators of virulence and induces transcriptional profiles consistent with GI niche adaptation^52^. Analysis of stool metagenomic datasets from publicly available cohorts of patients with CRC (*n* = 627) and healthy individuals (*n* = 619) indicates that the *eut* and *pdu* operons are significantly enriched in patients with CRC (two-sample *z*-test, two-tailed, *eut* *P* < 0.00001, *pdu* *P* < 0.00001; Extended Data Fig. 4a).

Motivated by the conservation of *eut* and *pdu* in *Fna* C2, and their absence in *Fna* C1, we assessed global transcriptomic responses of *Fna* cells following exposure to these intestinal-associated metabolites. RNA sequencing of representative *Fna* C1 and *Fna* C2 strains after exposure to EA or 1,2-PD indicated that both *Fna* clades have significant transcriptomic changes (Extended Data Fig. 4b–d and Supplementary Tables 11–15). As *Fna* C1 is deficient in both *eut* and *pdu*, we reasoned that significant transcriptomic changes following exposure to EA or 1,2-PD in *Fna* C1 would be independent of these operons. Thus, through a subtractive approach, we focused on differentially expressed genes (*t*-test, two-tailed, *P* < 0.05) in *Fna* C2 cells that were not differentially expressed (*t*-test, two-tailed, *P* > 0.05) in *Fna* C1 cells. Our results demonstrate that in *Fna* C2, *eut* and *pdu* genes are transcriptionally upregulated in response to EA and 1,2-PD, respectively (Fig. 3e,f).

Furthermore, *Fna* C2 cells exposed to EA or 1,2-PD significantly upregulated 13.02% of *Fna* C2-associated genes, including canonical *Fn* virulence factors. Although present in both clades, *radD* and *aim1* are upregulated in the presence of EA in *Fna* C2 but not *Fna* C1 cells (Fig. 3e). Virulence factors uniquely present in *Fna* C2 are additionally upregulated when *Fna* C2 cells are exposed to EA (*cmpA*, *fusolisin* and *fap2*) or 1,2-PD (*fap2*; Fig. 3e,f). The upregulation of *Fna* C2-associated genes and virulence factors known to be important for interactions with human epithelial cells suggests that following their transit to the human GI tract, sensing of these molecules could induce *Fna* C2 transcriptional profiles consistent with extra-oral niche adaptation.

**Extended Data Fig. 5:**
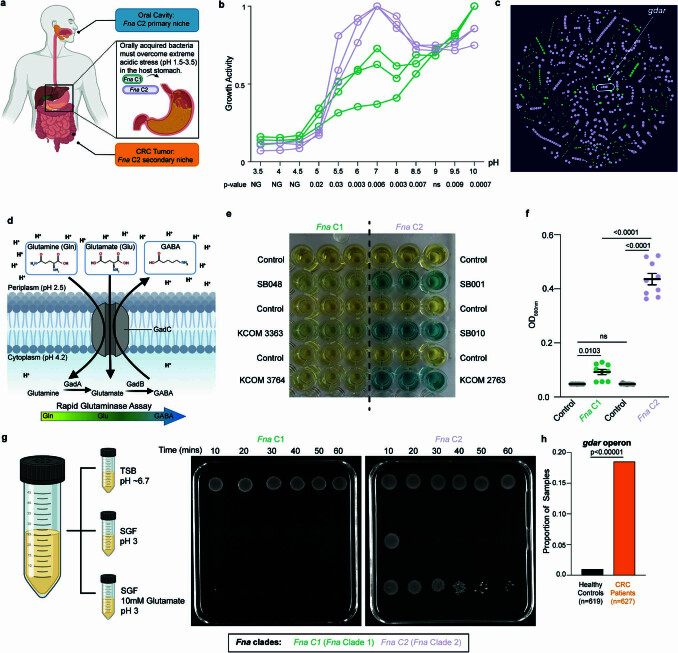
Differences in pH preference and acid resistance across *Fna* clades. **a**, Schematic of potential gastrointestinal route from the oral primary niche to the CRC tumor secondary niche. **b**, Plot indicates growth activity as measured in Biolog PM10 plates for representative *Fna* C1 (green) and *Fna* C2 (lavender) strains. Data is plotted as the normalized average across duplicates. Statistical analysis at each pH performed using Welch’s T-test, two-tailed. NG = no growth. **c**, PPanGGOLiN^51^ map of *Fna* pangenome. Each node represents a gene group, syntenic nodes represent neighboring genes, size indicates relative presence across *Fna* genomes, and color depicts elements in the *Fna* C1-associated accessory genome (green), and the *Fna* C2-associated accessory genome (lavender). White arrow indicates putative glutamate-dependent acid resistance (*gdar*) operon. **d**, Schematic depicts mechanism of GDAR acid resistance system. **e**, Qualitative and **f**, quantitative measurements of colorimetric assay measuring pH change indicative of conversion of glutamine to glutamate (yellow to green) and conversion of glutamate to γ-aminobutyric acid (GABA) (green to blue) in the presence of representative *Fna* C1 and *Fna* C2 strains. n = 3 technical triplicates of 3 biological replicates of 3 strains per *Fna* clade. Data is plotted as mean ± s.e.m and statistical analysis performed using an ANOVA. **g**, Schematic of experiment testing the effects of pH stress by exposure to simulated gastric fluid (SGF) at pH 3 or SGF supplemented with 10 mM glutamate at pH 3. Plates show resulting growth for a representative *Fna* C1 and a representative *Fna* C2 strain over the course of an hour exposure, as compared to tryptic soy broth (TSB) control at pH ~6.7. **h**, Bar plots demonstrate the proportion of stool metagenomic samples from patients with CRC (orange) or healthy controls (black) in which a putative *gdar* operon was detected. Statistical analysis performed using two sample Z test, two-tailed. Cohort sample sizes are indicated at the bottom of each panel. The graphics in **a,d,g** were created using BioRender.com.

This also led us to reconsider how *Fna* C2 might be translocating to extra-oral tumour niches. Previous studies suggest that oral fusobacteria travel to CRC tumours through the bloodstream during transient bacteremia caused by activities such as daily hygiene practices or dental procedures^20^. Our identification of transcriptionally active *eut* and *pdu* operons suggests that direct descent through the GI tract, with subsequent infiltration of CRC tumours through the lumen, may be an additional pathway used by *Fna* C2. Yet, for GI transit to be a viable route of dissemination, *Fna* C2 would need to overcome the deleterious effects of extreme acid stress encountered in the stomach (pH 1.5–3.5; Extended Data Fig. 5a). Assessing the preferential growth pH, we observed that both *Fna* clades are sensitive to pH below 4.5 (Extended Data Fig. 5b). From pH 5.5 to 8.5, *Fna* C2 strains had a significantly higher level of growth activity compared to *Fna* C1, with maximum growth activity at pH 7 (Extended Data Fig. 5b). Under basic conditions (pH 9.5–10), *Fna* C1 strains had significantly higher growth activity compared to *Fna* C2, with maximum growth activity at pH 10 (Extended Data Fig. 5b). Pangenome analysis also revealed a putative glutamate-dependent acid resistance (GDAR) system conserved across all *Fna* C2, but absent in *Fna* C1 (Extended Data Fig. 5c and Supplementary Table 10). The GDAR system, found in pathogenic and commensal gut bacteria, is one of the most potent acid resistance mechanisms^53^, with glutamate being the only component necessary for the system to operate at pH ≤3 (Extended Data Fig. 5d). Using a colorimetric pH change assay, we tested the conversion of glutamine through glutamate into γ-aminobutyric acid in the presence of *Fna* C1 and *Fna* C2 and found that the level of this conversion is significantly higher in the presence of *Fna* C2 strains (Extended Data Fig. 5e,f).

To further mimic effects of pH stress during gastric transit, we exposed *Fna* clades to simulated gastric fluid at pH 3. Both were non-viable after 10 min of exposure to simulated gastric fluid. However, in the presence of supplemented glutamate, *Fna* C2 survived for an extended period (about 60 min), which was not observed for *Fna* C1 lacking GDAR (Extended Data Fig. 5g). Analysis of stool metagenomic datasets indicated that *gdar* operons are significantly enriched in patients with CRC compared to healthy individuals (two-sample *z*-test, two-tailed, *P* < 0.00001; Extended Data Fig. 5h). Thus, in addition to active *eut* and *pdu* systems, differences in pH preference and acid resistance mechanisms may contribute to the ability of *Fna* C2 to access the GI and tumour niches.

## *Fna* C2 affects intestinal tumorigenesis

**Fig. 4:**
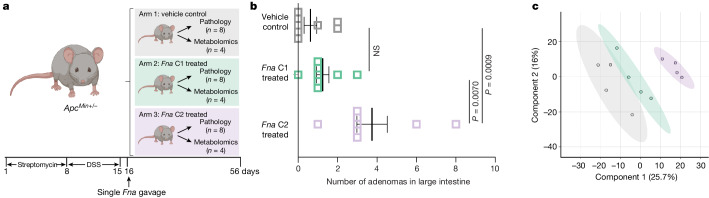
*Fna* C2 impact on intestinal tumorigenesis and metabolism. **a**, A schematic of the study with 6–8-week-old *Apc*^*Min+/−*^ mice receiving streptomycin and dextran sodium sulfate (DSS) treatment to alter the native microbiome and induce colitis, respectively. Mice were orally gavaged with vehicle control (arm 1) or a mix of three representative *Fna* C1 (arm 2) and *Fna* C2 (arm 3) strains. A strain mix was used to capture a higher proportion of *Fna* clade-specific accessory genes (Extended Data Fig. 2d). The mice were monitored until the end-point at 6 weeks post-gavage when they reached 15–17 weeks of age. **b**, A plot indicating the number of adenomas in the large intestine by treatment arm (vehicle control (grey); *Fna* C1 treated (green); *Fna* C2 treated (lavender); *n* = 8 mice per arm). The data are plotted as mean ± s.e.m. The statistical analysis was carried out using one-way ANOVA. **c**, Partial least squares discriminant analysis of detected intestinal metabolites (*n* = 1,296). The colours represent the treatment arm (vehicle control (grey); *Fna* C1 treated (green); *Fna* C2 treated (lavender)). The graphics in **a** were created using BioRender.com. Source Data

**Extended Data Fig. 6:**
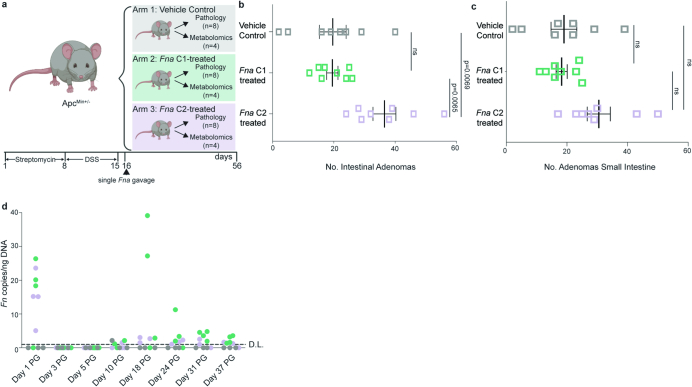
Intestinal adenoma burden and fecal *Fusobacterium* load in *Fna* treated mice. **a**, Schematic of study with Apc^Min+/−^ mice orally gavaged with vehicle control (Arm 1) or representative *Fna* C1 (Arm 2) and *Fna* C2 (Arm 3) strains post streptomycin and dextran sodium sulfate (DSS) to clear the native microbiome and induce colitis, respectively. **b**-**c**, Plots indicate the number of adenomas in **b**, the small and large intestines combined and **c**, the small intestine by treatment arm, vehicle control (grey), *Fna* C1-treated (green), or *Fna* C2-treated (lavender). Data is plotted as mean ± s.e.m; n = 8 mice per treatment arm. Statistical analysis performed using one-way ANOVA. **d**, *Fusobacterium*-targeted qPCR was performed on fecal pellets from one-day post-gavage (PG) to study endpoint. *Fn* presence in each sample is plotted as *Fn* copies per ng of fecal DNA with a detection limit (D.L.) of 1 *Fn* copy. Each sample was run in triplicate, and samples with ≥2 duplicates with detectable signal are included. The graphics in **a** were created using BioRender.com. Source Data

As *Fna* C2-enriched gene clusters were predominantly associated with enhanced metabolic potential (Extended Data Fig. 3e and Supplementary Table 10), we sought to determine whether *Fna* treatment of the dextran sodium sulfate-induced colitis *Apc*^*Min+/−*^ mouse model^54^ of CRC affected intestinal tumorigenesis and metabolic pathways in vivo (Fig. 4a). To capture a higher proportion of *Fna* clade-specific accessory genes (Extended Data Fig. 2d), a mix of three representative strains for each clade was used. Following the administration of a single oral gavage of *Fna* C1 mix, *Fna* C2 mix or vehicle control, we observed a significant increase in the number of intestinal adenomas in *Fna* C2-treated mice compared to both *Fna* C1 and vehicle control independently (Extended Data Fig. 6a,b; analysis of variance (ANOVA), *P* = 0.0065 and *P* = 0.0069, respectively), specifically in the large intestine (Fig. 4b; ANOVA, *P* = 0.0070 and *P* = 0.0009, respectively; Extended Data Fig. 6c). There was no significant difference in adenoma burden between *Fna* C1 treatment and vehicle control mice. Low-level *Fn* was inconsistently detected during the course of the study (Extended Data Fig. 6d). We carried out liquid chromatography–mass spectrometry global metabolomics on intestinal tissue from each treatment arm for comparative metabolite analysis (Supplementary Table 16). Partial least squares discriminant analysis of measured intestinal metabolites demonstrated that *Fna* C2-treated mice formed a distinct cluster away from other treatment arms, suggesting a differential metabolic profile. However, intestinal metabolites from *Fna* C1-treated and vehicle control mice had more similar metabolite profiles, clustering together (Fig. 4c).

**Extended Data Fig. 7:**
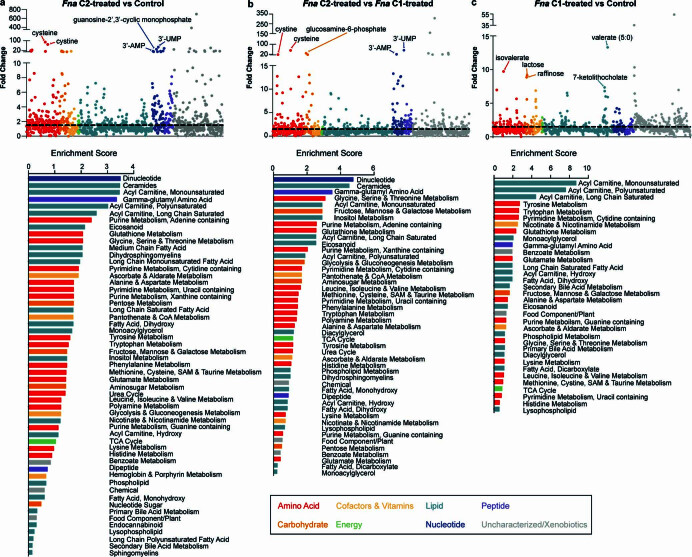
Intestinal metabolite changes in *Fna* treated mice. Scatter plots shows fold change for 1,296 detected metabolites from **a**, *Fna* C2 treated mice compared to control, **b**, *Fna* C2 treated mice compared to *Fna* C1 treated mice, and **c**, *Fna* C1 treated mice compared to control colored by metabolic categorization. Top five characterized metabolites are labeled. Plots below indicate the pathway enrichment score all enriched pathways. Bars are colored by metabolic categorization.

**Extended Data Fig. 8:**
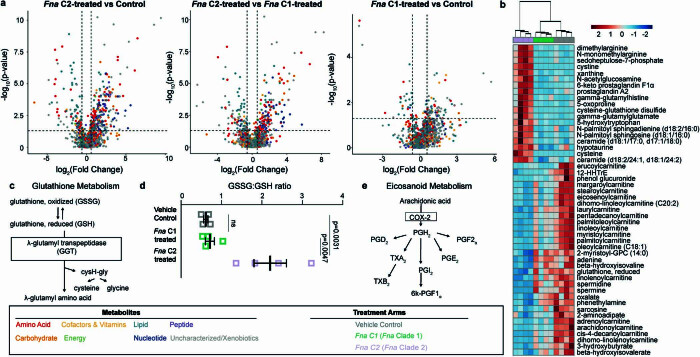
Altered metabolites in *Fna* treated mice. **a**, Plots show the log2-transformed fold change in metabolite level between treatment arms by the -log10(p-value). Points indicate individual metabolites colored by their metabolic categorization. Dotted lines indicate the threshold of significant gene expression, defined as log2-transformed fold change ≥ 0.58 and ≤ −0.58 (vertical lines) and a -log10(p-value) ≥ 1.30 (horizontal line). Statistical analysis performed on natural log-transformed values using one-sided T-test. **b**, Clustered heatmap of top fifty metabolites across study arms. Dendrogram groups individual samples by similarity of metabolite profile. Bar color for each sample indicates treatment arm, vehicle control (grey), *Fna* C1-treated (green), or *Fna* C2-treated (lavender). **c**, Schematic of glutathione metabolic pathway. **d**, Plot demonstrates the ratio between oxidized (GSSG) and reduced (GSH) glutathione levels for each treatment arm, vehicle control (grey), *Fna* C1-treated (green), or *Fna* C2-treated (lavender); *n* = 4 mice per arm. Data is plotted as mean ± s.e.m. Statistical analysis performed using one-way ANOVA. **e**, Schematic of eicosanoid metabolic pathway.

**Extended Data Fig. 9:**
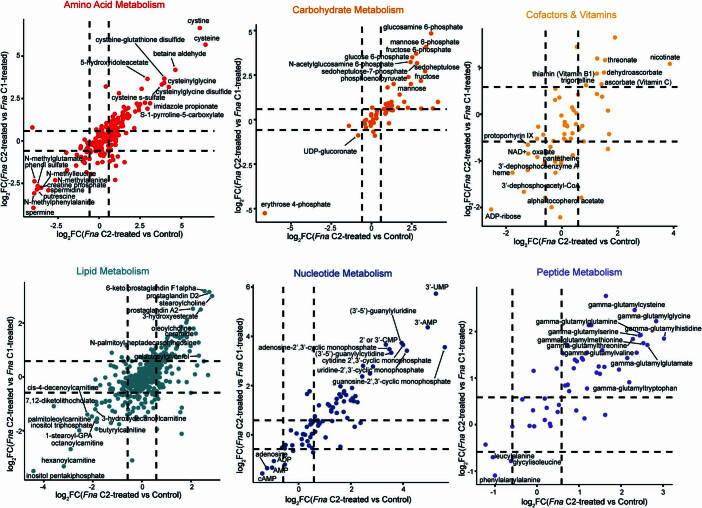
Significantly altered intestinal metabolites in *Fna* C2-treated mice. Plots of individual, characterized metabolites, with metabolic categories having > 10 significantly altered metabolites shown. Each plot shows the log2-transformed log change between *Fna* C2-treated mice compared to *Fna* C1-treated mice versus the log2-transformed log change between *Fna* C2-treated mice compared to control mice. Dashed lines indicate the threshold of significantly altered metabolites, defined as log2-transformed fold change (FC) ≥ 0.58 and ≤ −0.58. Upper right quadrant indicates metabolites that are significantly elevated in *Fna* C2-treated mice as compared to both *Fna* C1-treated and control mice. Lower left quadrant indicates metabolites that are significantly lower in *Fna* C2-treated mice as compared to both *Fna* C1-treated and control mice. Top metabolites in each of these quadrants are labeled.

Of 1,296 metabolites measured (Extended Data Fig. 7a–c and Supplementary Table 16), comparative analysis demonstrated a significant enrichment in glutathione metabolism and γ-glutamyl amino acid pathways in *Fna* C2-treated mice, compared to both *Fna* C1-treated and vehicle control mice (Extended Data Figs. 7a–c, 8a and 9 and Supplementary Table 17). Specifically, we observed a significant increase in the levels of precursors to γ-glutamyl-cysteinyl-glycine (GSH) synthesis, including cysteine and γ-glutamylcysteine, decreased levels of glutathione in its reduced form (GSH) and significantly higher levels of the GSH degradation product 5-oxoproline (Extended Data Figs. 8b and 9 and Supplementary Table 16). Consistent with γ-glutamyl amino acid generation from reduced glutathione in the presence of γ-glutamyl transpeptidase, increased levels of γ-glutamyl amino acids and cysteine, glycine and cysteinyl-glycine were also observed (Extended Data Fig. 8b,c and Supplementary Table 16). GSH deficiency or an elevated ratio of oxidized (GSSG) to reduced (GSH) forms of glutathione increases the vulnerability of mammalian cells to oxidative stress, inflammation and tumour progression^55^. The GSSG/GSH ratio of *Fna* C2-treated mice significantly increased by 3.5- and 3.0-fold compared to the control and *Fna* C1-treated groups respectively, suggesting increased oxidative stress (Extended Data Fig. 8d; one-way ANOVA, *P* = 0.0031 and *P* = 0.0047, respectively). Studies have demonstrated that metabolism of GSH by γ-glutamyl transpeptidase can exert pro-oxidant effects. In cancer cells, this is a source of endogenous reactive oxygen species that can facilitate persistent oxidative stress and contribute to genomic instability^56,57^. Consistent with this, significantly increased levels of other markers of oxidative stress including cystine and cysteine-glutathione disulfide and significantly decreased levels of polyamines capable of scavenging reactive oxygen species, including putrescine, spermidine and spermine, were observed in *Fna C2*-treated mice (Extended Data Figs. 8b and 9). Notably, recent work from our group demonstrated that *Fusobacterium* was predominantly associated with epithelial cells harbouring severe chromosomal abnormalities^10^, one of the most common forms of genomic instability in cancer.

In addition to their role in combating oxidative stress, polyamines are able to suppress inflammation through inhibition of macrophage cytokine synthesis^58^. Consistent with increased inflammation, significantly higher levels of *N*-monomethylarginine and dimethylarginine were observed in *Fna* C2-treated mice compared to other treatment arms (Extended Data Fig. 8b). Both of these metabolites inhibit the synthesis of the anti-inflammatory agent nitric oxide. Furthermore, we observed significantly higher levels of pro-inflammatory prostaglandins and ceramides, including prostaglandin A2, *N*-palmitoyl-sphingosine and *N*-palmitoyl-sphingadienine (Extended Data Fig. 8b). Ceramides can also be metabolized by cancer cells to reduce tumour cell apoptosis and proliferation^59^. Other metabolites that promote cancer cell proliferation and metastasis across a range of cancers include eicosanoids, which are similarly significantly increased in *Fna* C2-treated mice compared to *Fna* C1-treated or vehicle control mice (Extended Data Figs. 7a–c and 8e and Supplementary Table 17). This included increased levels of 6-keto prostaglandin F1-α (Extended Data Fig. 8b) through COX2 (also known as PTGS2) metabolism of arachidonic acid. Notably, *COX2* was previously reported to be one of the most upregulated genes in *Fn*-associated human colorectal tumours^3^. Overall, our results demonstrate the ability of *Fna* C2, but not *Fna* C1, to metabolically affect the intestinal milieu towards pro-oncogenic conditions.

## *Fna* C2 enrichment in human CRC cohorts

**Fig. 5:**
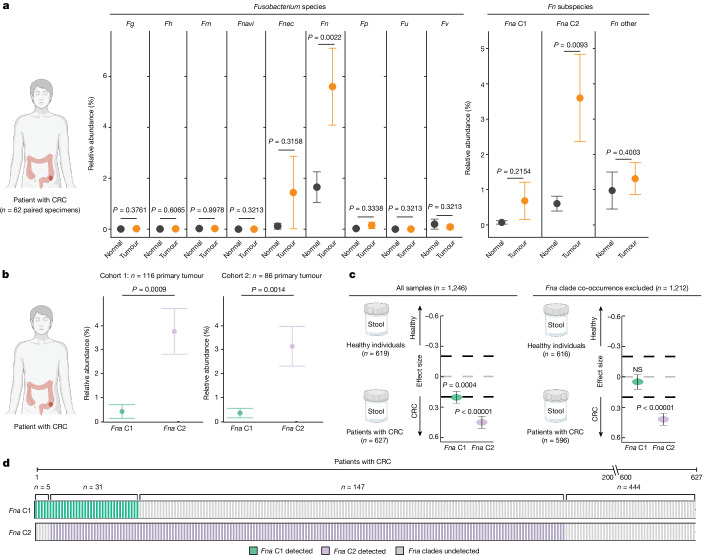
*Fn* in human tissue microbiome and stool metagenomic specimens. **a**, Plots showing the relative abundance for *Fusobacterium* species (*Fg*, *F.* *gonidiaformans*; *Fh*, *F.* *hwasookii*; *Fm*, *F.* *mortiferum*; *Fnavi*, *F.* *naviforme*; left plot), and *Fn* subspecies and *Fna* clades (right plot) using microbial 16S rRNA gene sequencing of paired tumour (orange) and normal adjacent (black) tissue (*n* = 62 patients with CRC). Amplicon sequence variants were used to obtain *Fna* clade resolution (Extended Data Fig. 10 and Supplementary Table 8). The data are plotted as mean ± s.e.m. The statistical analysis was carried out using one-sided *t*-test, paired. **b**, Plots showing the relative abundance for *Fna* C1 (green) and *Fna* C2 (lavender) within patient primary colorectal tumour tissue from two independent cohorts (cohort 1 (*n* = 116) this study; cohort 2 (*n* = 86) BioProject PRJNA362951). The data are plotted as mean ± s.e.m. The statistical analysis was carried out using one-sided *t*-test, paired. **c**, *Fna* C1 and *Fna* C2 detection in stool metagenomic data from patients with CRC and healthy individuals. The left plot shows the pooled effect sizes for *Fna* C1 (green) and *Fna* C2 (lavender) calculated using a meta-analysis of standardized mean differences and a random-effects model on MetaPhlAn4 (ref. ^63^) species-level genome bin abundances on all CRC samples (*n* = 627) and samples from healthy individual (*n* = 619). The right plot shows the effect sizes for *Fna* C1 and *Fna* C2 calculated using the same approach, but here samples in which *Fna* C1 co-occurred with *Fna* C2 were excluded. The data are plotted as mean ± s.e.m. The statistical significance was assessed by a Wald test, two-sided. All *P* values are corrected using the Benjamini–Yakuteli method. **d**, *Fna* C1 and *Fna* C2 presence in stool metagenomes of patients with CRC. The bars indicate individual stool samples from patients with CRC (*n* = 627) and are coloured by *Fna* C1 and *Fna* C2 detection (*Fna* C1 detected (green); *Fna* C2 detected (lavender); *Fna* undetected (grey)). The lower brackets indicate the number of stool samples in which *Fna* C1 occurred independently (*n* = 5), *Fna* C2 occurred independently (*n* = 147), *Fna* clades co-occurred (*n* = 31) or *Fna* clades were not detected (*n* = 444). The graphics in **a**–**c** were created using BioRender.com.

As *Fna* C2 strains are both significantly enriched in the CRC niche (Fig. 2f) and increase intestinal tumorigenesis in our mouse model compared to *Fna* C1 (Fig. 4b and Extended Data Fig. 6a–c), we next sought to determine the prevalence and abundance of these *Fna* clades in human tissue and stool specimens through culture-independent approaches. We carried out bacterial 16S rRNA gene sequencing on resected tumour tissue from 116 patients with treatment-naive CRC (CRC cohort 1) and on adjacent normal tissue from 62 of these patients (Supplementary Table 18). Comparing the percentage relative abundance of different *Fusobacterium* species between paired tumour and adjacent normal tissue (*n* = 62 patients), we observed that *Fn* was the only *Fusobacterium* species significantly enriched in tumour tissue compared to adjacent normal (Fig. 5a and Supplementary Table 19), supportive of previous reports^2,60^ (*t*-test, paired, *P* = 0.0022). However, using the *Fna* clade-specific amplicon sequence variants to resolve *Fn* to a higher taxonomic resolution that includes *Fna* C1, *Fna* C2 and non-*Fna* subspecies of *Fn*, we demonstrate that only *Fna* C2 is significantly enriched in tumour compared to paired normal tissue (Fig. 5a, and Supplementary Tables 8 and 19; *t*-test, paired, *P* = 0.0093). As neither *Fna* C1 nor non-*Fna* subspecies of *Fn* are significantly enriched, this suggests that it is specifically *Fna* C2 that is driving the previously reported enrichment of *Fn* in human CRC tumours. Furthermore, across two independent patient cohorts (CRC cohort 1 *n* = 116 and CRC cohort 2 *n* = 86), we demonstrate that within CRC tumour tissue, *Fna* C2 is significantly enriched compared to *Fna* C1 (Fig. 5b and Supplementary Tables 20 and 21; *t*-test, paired, cohort 1 *P* = 0.0009, cohort 2 *P* = 0.0014), supporting our observations at the *Fna* strain level (Fig. 2f).

**Extended Data Fig. 10:**
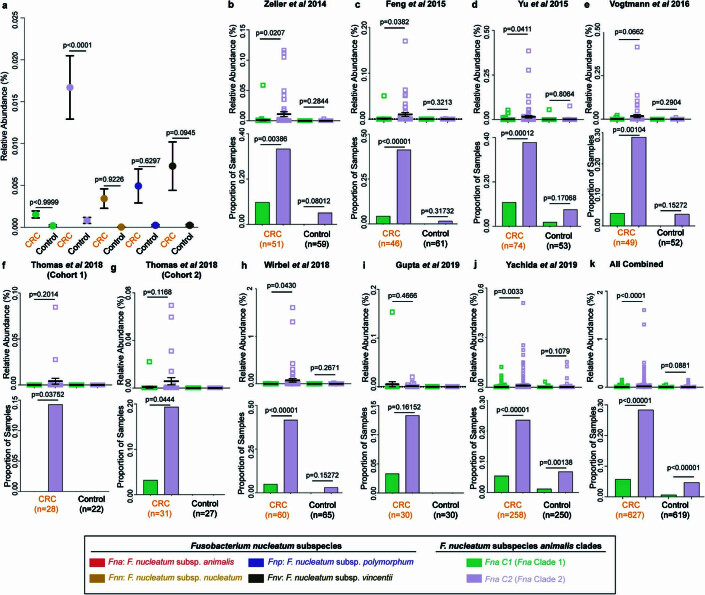
Detection of *Fn* subspecies and *Fna* clades in human stool metagenomes. Detection of **a**, *Fn* subspecies (*Fnn*, *Fnv*, *Fnp*) and **a**-**k**, *Fna* clades (*Fna* C1 and *Fna* C2) in stool metagenomic data from previously published independent cohorts from patients with CRC and healthy controls. **a**, Plot shows the percent relative abundance of each *Fn* subspecies and *Fna* clade in stool samples from patients with CRC (n = 627) or healthy controls (n = 619). Data is plotted as mean ± s.e.m and statistical analysis performed using a one-way ANOVA. **b**-**k**, Samples are plotted both by (**b**-**k**) individual cohorts and (**k**) pooled results. In each panel, top plot shows the percent relative abundances of *Fna* C1 (green) and *Fna* C2 (lavender) in each sample. Data is plotted as mean ± s.e.m and statistical analysis performed using Welch’s T-test, paired. Bottom plot demonstrates the proportion of stool samples from patients with CRC and the proportion of stool samples from healthy controls in which *Fna* C1 and *Fna* C2 were detected. Data is plotted as mean + s.d. and statistical analysis performed using two sample Z test, two-tailed. Cohort sample sizes are indicated at the bottom of each panel.

**Extended Data Fig. 11:**
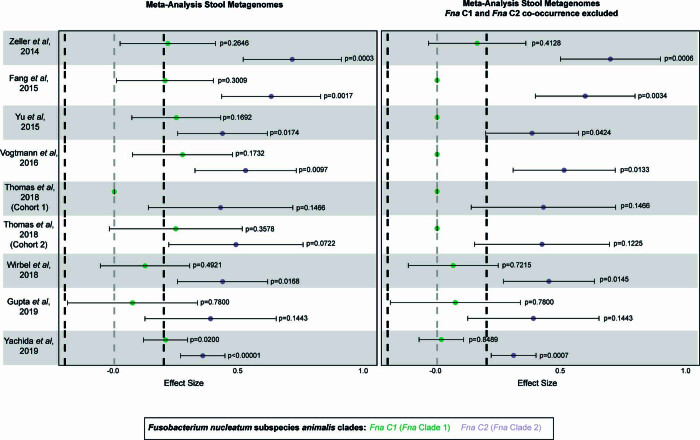
Meta-analysis of *Fna* in human stool metagenomes. *Fna* C1 and *Fna* C2 detection in stool metagenomic data from previously published independent cohorts from patients with CRC and healthy controls plotted by individual cohorts. Left plot shows the effect sizes for *Fna* C1 and *Fna* C2 calculated across all samples (CRC n = 627, healthy control n = 619) using a meta-analysis of standardized mean differences and a random effects model on MetaPhlAn4^63^ species-level genome bins (SGB) abundances. Right plot shows the effect sizes for *Fna* C1 and *Fna* C2 calculated using the same approach but with samples where *Fna* C1 co-occurred with *Fna* C2 excluded (CRC n = 596, healthy control n = 616). Data is plotted as mean ± s.e.m. Statistical significance assessed by Wald test, two-sided. All p-values are corrected via the Benjamini-Yakuteli method.

We next sought to determine whether the prevalence of *Fna* clades differed between patients with CRC and healthy individuals. To do so, we analysed stool metagenomic datasets from publicly available cohorts of patients with CRC (*n* = 627) and healthy individuals (*n* = 619; Extended Data Fig. 10 and Supplementary Table 22). *Fna* was detected in 29.2% of stool samples from patients with CRC and 4.8% of stool samples from healthy individuals (Supplementary Table 23). Meta-analysis of standardized mean differences by a random-effects model for *Fna* C1 and *Fna* C2 demonstrated that both *Fna* clades have a significant pooled effect size associated with CRC (*Fna* C1 effect size = 0.21, 95% confidence interval (0.09, 0.32), *P* = 4.45 × 10^−4^; *Fna* C2 effect size = 0.45, 95% confidence interval (0.34, 0.56), *P* = 5.55 × 10^−15^; Fig. 5c, Extended Data Fig. 11 and Supplementary Table 24). However, the effect size for *Fna* C2 was larger than that for *Fna* C1. Notably, in the absence of *Fna* C2 co-occurrence, *Fna* C1 was not significantly associated with CRC (Fig. 5c, Extended Data Fig. 11 and Supplementary Table 25). Although synergistic interactions between CRC-enriched microbes have previously been reported^61^, it is not clear whether *Fna* C1 co-occurrence with *Fna* C2 results in a compounding pathogenic effect. Similar to our observation in CRC tumour tissue (Supplementary Tables 20 and 21), our data show that *Fna* C2 is more prevalent and abundant in the stool of patients with CRC than *Fna* C1 (Fig. 5d and Extended Data Fig. 10) and is furthermore the only *Fn* subgroup significantly enriched in the stool of patients with CRC compared to healthy individuals (Extended Data Fig. 10a). These culture-independent human specimen analyses support our strain-level genomic discovery that *Fna* C2 is the dominant CRC-associated *Fna* clade (Fig. 2). This further highlights the significance of our in vitro (Fig. 3) and in vivo (Fig. 4) findings demonstrating the increased virulence and tumorigenic potential of *Fna* C2 compared to *Fna* C1.

## Discussion

Advances in next-generation sequencing have revealed the presence of bacterial communities within human tumour tissues. A key challenge for cancer microbiome research is to move beyond the characterization of microbial composition in tumours towards functional studies that determine whether, and how, these microbes are contributing to disease. In CRC, *Fn* gained early and continued attention owing to the fact that this bacterium was rarely detected in the lower GI tract of healthy individuals^1^, yet enriched within the CRC tumour microbiome^2,60^. *Fn* species are normal members of the human oral microbiota, and strains from the oral cavity are thought to seed CRC tumours^19,20^. However, the noted genetic and phenotypic heterogeneity^12–15^ of *Fn* led to an open question of whether *Fn* strains that colonize and dominate human tumours harbour distinct genetic attributes that contribute to CRC initiation or progression. Through large-scale culturing, sequencing and comparative genomic analyses of human CRC and non-cancer oral *Fn* strains, we revealed the distinct CRC-enriched genetic factors of *Fn*. Further, we identified that these CRC-enriched factors were predominantly present within a specific clade of *Fna*. This was mirrored by our discovery that *Fna* is bifurcated into two distinct clades: *Fna* C1, which is largely restricted to the oral cavity, and *Fna* C2, which dominates the human CRC tumour niche. Notably, only *Fna* C2 induced tumours and altered intestinal metabolism towards increased oxidative stress within a CRC animal model. Further, comparative genomic analysis between *Fna* clades revealed the genetic elements that cumulatively engender the pathoadaptation of *Fna C2* to the CRC niche. Given the power of using *Fna* C1 as a comparative group for *Fna* C2, we created an interactive website to enable the exploration of *Fna* pangenomic datasets designated The Fusobacterium Pangenome Atlas at https://fredhutch.github.io/fusopangea/. Collectively, this work demonstrates that *Fna* C2 is a highly virulent subgroup of *Fn* that should be the primary focus for mechanistic studies and therapeutic drug design in CRC.

## Methods

### *Fusobacterium* strain isolation from tumour tissue from patients with CRC

*Fusobacterium* strains were isolated from CRC tumour tissue specimens from patients from North America and Europe as previously described^6^. Briefly, tissue sections were minced with a scalpel, and spread plated on selective fastidious anaerobe agar (FAA) plates (Oxoid, Thermo Fisher Scientific) supplemented with 7% or 10% defibrinated horse blood (DHB; Lampire Biological Laboratories, Fisher Scientific) with josamycin, vancomycin and norfloxacin at 3, 4 and 1 μg ml^−1^, respectively (Sigma Aldrich). Plates were incubated at 37 °C in anaerobic conditions (AnaeroGen Gas Generating Systems, Oxoid, Thermo Fisher Scientific) and inspected for growth every 2 days. Colonies were picked and streak purified, and colony PCR was carried out on selected bacterial colonies as previously described^6^ with 16S rRNA gene universal primers (342F and 1492R). Colony PCR products were sent for Sanger sequencing, and BLASTn analysis of trace sequences was used to confirm bacterial species identity. Cultures were suspended in tryptic soy broth (TSB) and 40% glycerol and stored at −80 °C.

### *Fusobacterium* strain isolation from Korean Collection for Oral Microbiology and ATCC ampoules

*Fusobacterium* strains from the Korean Collection for Oral Microbiology (KCOM) collection were isolated from the oral cavity as previously described^44^. Strains from the ATCC and KCOM repositories were grown from ampoules on Schaedler agar plates supplemented with vitamin K_1_ and 5% defibrinated sheep blood (Becton Dickinson) and FAA plates (Oxoid, Thermo Fisher Scientific) supplemented with 7% DHB (Lampire Biological Laboratories, Fisher Scientific). Plates were incubated at 37 °C in a Bactron600 anaerobic chamber (Sheldon Manufacturing) for 5–7 days. Cultures were suspended in Schaedler broth with vitamin K_1_ and 30% glycerol and stored at −80 °C.

### High molecular weight genomic DNA extraction

*Fusobacterium* strains were cultured under anaerobic conditions at 37 °C (AnaeroGen Gas Generating Systems, Oxoid, Thermo Fisher Scientific) for 48–72 h on FAA plates (Oxoid, Thermo Fisher Scientific) supplemented with 10% DHB (Lampire Biological Laboratories, Fisher Scientific) and plates for CRC-associated strains were further supplemented with josamycin, vancomycin and norfloxacin at 3, 4 and 1 μg ml^−1^, respectively (Sigma Aldrich). High molecular weight genomic DNA was extracted using the MasterPure Gram Positive DNA Purification Kit (Epicentre, Lucigen). Cells from two plates were resuspended in 1.5 ml 1× PBS and collected by centrifugation. Pellets were processed according to the manufacturer’s instructions, modified by doubling all reagent volumes and removing vortexing steps to prevent DNA shearing. High molecular weight genomic DNA was quantified using a Qubit fluorometer (Thermo Fisher Scientific).

### PacBio single-molecule real-time sequencing and genome assembly

Single-molecule real-time sequencing^18^ was carried out on a PacBio Sequel instrument (Pacific Biosciences) or a PacBio Sequel II instrument (Pacific Biosciences) at the University of Minnesota Genomics Center. Sequencing reads were processed using Microbial Assembly pipeline within Pacific Biosciences’ SMRTAnalysis pipeline v.9.0.0.92188. Additional assembly was carried out using Flye assembler v.2.8 as needed (https://github.com/fenderglass/Flye).

### *Fusobacterium* species typing

*Fusobacterium* genomes were subtyped to the species level and *Fn* genomes were further subtyped to the subspecies level on the basis of a cumulative score of individual marker genes. Marker genes previously used for *Fusobacterium* typing were used: the 16S rRNA gene, *rpoB* and a zinc metalloprotease gene^30^. From each complete, closed genome, its species or subspecies classification was first analysed by all three marker genes individually. Each marker gene was isolated and analysed using BLASTn, with the top hit by percentage identity noted. For each possible species or subspecies, a confidence score was calculated as the number of concordant subspecies results divided by the number of marker genes present. For each genome, its final classification was determined by the highest confidence score. Results for this analysis are noted in Supplementary Table 1. Phylogenetic classifications were further tested using GTDB-Tk (ref. ^64^; https://github.com/Ecogenomics/GTDBTk) as listed in Supplementary Table 2.

### Pangenomic analyses

Pangenome analysis was carried out using the Anvi’o workflow^21^, the PPanGGOLiN tool^51^ and the GiG-map tool (https://github.com/FredHutch/gig-map) to characterize the *Fn* pangenome across 135 *Fn* genomes, and to characterize the *Fna* pangenome across 51 *Fna* genomes. For *Fn* genomes, Anvi’o thresholds were set to a minbit of 0.9 and an MCL of 2, and PPanGGOLiN thresholds were set to 90% identity and 90% coverage. For *Fna* genomes, Anvi’o thresholds were set to a minbit of 0.9 and an MCL of 7, and PPanGGOLiN thresholds were set to 90% identity and 90% coverage. For both genome sets, GiG-map was run with default settings. PPanGGOLiN’s alignment feature was used to map resulting Anvi’o gene clusters to their corresponding PPanGGOLiN nodes. To assess the size of the pangenome as the number of sampled genomes increases, the *Fn* and *Fna* Anvi’o-derived pangenomes were independently sampled for combinations up to 10,000 or otherwise randomly subsampled 10,000 times from 1 to 135 genomes and 1 to 75 genomes, respectively. This approach was subset by niche and clade as appropriate.

### Genomic dendrograms

Individual gene and protein sequences were aligned through MEGA X (ref. ^65^) using the MUSCLE clustering algorithm from which a maximum-likelihood dendrogram was generated. kSNP3 (ref. ^45^) with a *k-*mer size of 13, resulting in a fraction of core *k*-mers of 0.217, was used to generate a maximum-likelihood phylogeny of the 135 *Fn* genomes in our collection. Final images were generated using the interactive tree of life tool, v.5 (ref. ^66^).

### Identification of *Fn* canonical virulence factors

To query the presence of canonical *Fn* virulence genes in our collection of *Fn* genomes, we used the Operon Contextualization Across Prokaryotes to Uncover Synteny tool (https://github.com/FredHutch/octapus) with a minimum percentage identity threshold of 60%.

### Identification of *Fn* genetic defence systems and prophage

The presence of innate bacterial defence systems was queried using the Prokaryotic Antiviral Defense Locator^67^ and intact prophage presence was analysed using the Phage Search Tool Enhanced Release^68,69^ tools.

### PCA

PCA of *Fn* Anvi’o-derived gene content was carried out on a gene cluster presence–absence matrix using the R prcomp function in the stats package, v.3.6.2. PCA of *Fna* methylated nucleotide motifs was carried out on a methylated motif presence–absence matrix (Supplementary Table 7) using the PCA function in the R factoextra package, v.1.0.7.

### *Fn* and HCT116 co-culture assays

The human colon cancer epithelial cell line HCT116 was purchased from ATCC. The cell line was not authenticated. Mycoplasma testing was carried out using the MycoProbe Mycoplasma Detection Kit (R&D Systems). HCT116 cells were cultured in McCoys 5A with l-glutamine (Corning) supplemented with 10% (v/v) fetal bovine serum (Sigma) and incubated at 37 °C in 5% CO_2_. HCT116 cells were seeded at 1.25 × 10^6^ cells per well into 6-well plates with a glass coverslip at the bottom of each well (Nunclon Delta Surface, Thermo Scientific) and allowed to adhere for 16 h. Resuspended cultures of *Fna* C1 (SB048, KCOM 3363 and KCOM 3764) and *Fna* C2 (SB001, SB010 and KCOM 2763) strains were prepared in McCoys. Bacterial membranes were stained with 5 µg ml^−1^ FM 4-64FX (Molecular Probes). Each bacterial strain was co-incubated with HCT116 cells in wells at a multiplicity of infection of 100:1. These bacterial–eukaryotic co-cultures were incubated for 3 h at 37 °C in 5% CO_2_. Bacterial viability was assessed at time (*T*) = 0, *T* = 1.5 and *T* = 3 h by preparing serial dilutions for each strain and plating 50 µl of each dilution on FAA plates (Oxoid, Thermo Fisher Scientific) supplemented with 10% DHB (Hemostat, Fisher Scientific). Plates were incubated at 37 °C in a Bactron600 anaerobic chamber (Sheldon Manufacturing) for 2 days until colonies were counted. After incubation, wells were washed four times with PBS with gentle swirling to remove unattached bacterial and HCT116 cells. Cells were fixed in 4% paraformaldehyde in PBS for 30 min at room temperature. Following fixation, cells were washed three times in PBS and then permeabilized with 0.2% (v/v) Triton X-100 in PBS for 4 min at room temperature. Cells were washed three times in PBS and then stained for 20 min at room temperature with two drops per millilitre of NucBlue Fixed Cell Stain ReadyProbes (Invitrogen) and ActinGreen 488 ReadyProbes (Invitrogen) to stain DNA and actin, respectively. A dissecting microscope was used to visually confirm that cells remained on the coverslips after processing. Samples were viewed with a Leica SP8 confocal laser scanning microscope (Leica) for image acquisition. Three *z*-stacks of each co-culture were taken using a 63× oil lens and the following parameters: 1,024 × 1,024 resolution, pixel size 100.21 nm, speed 600, zoom factor 1.9 and *z*-step 0.3 mm.

### Computational analysis to determine intracellular *Fn*

Confocal *z*-stacks from bacterial–eukaryotic co-cultures of HCT116 cells co-incubated with *Fna* C1 (SB048, KCOM 3363 and KCOM 3764) or *Fna* C2 (SB001, SB010 and KCOM 2763) strains were imported into Imaris. All measurements were carried out on three different *z*-stacks per biological replicate, with three biological replicates. In Imaris, the bacterial surface volumes were created using the fluorescence of the FM 4-64FX membrane stain (surface detail 0.223 mm, background subtraction using diameter of largest sphere of 0.5 mm). The eukaryotic cell detection tool was used to define and ID cells using the nuclear stain and the actin stain. The nuclei were split by seed points. The detected eukaryotic cells were exported to create a cell surface mask. To define intracellular bacterial cells, the bacterial surface was classified by the shortest distance to the eukaryotic cell surface (min to −0.0000001 distance to eukaryotic cell membrane). This new classification was exported as a new ‘intracellular bacterial cell’ surface. To assess the number of eukaryotic cells with intracellular bacteria, the number of objects defined by the eukaryotic cell surface mask with internal objects defined by the ‘intracellular bacterial cell’ surface mask was counted. Statistical comparison of the percentage of HCT116 cells with intracellular *Fna* bacterial cells by *Fna* clade was carried out by applying a Welch’s *t*-test using GraphPad Prism v.7.0 software (GraphPad Software).

### Cell length and width measurements

*Fna* C1 and *Fna* C2 strain cell dimensions were measured using Fiji with the Bioformats Plugin (required to import Leica.lif files). First, the scale of the image was set by going to Analyze, then Set Scale, and then Set 1 mm to equal 9.979 pixels (pixel size 100.21 nm). Measurements were then captured using the freehand straight-line tool from the brightest point on each cell membrane stain. Statistical comparison of cell lengths and cell width by *Fna* clade was carried out by applying a Welch’s *t*-test using GraphPad Prism v.7.0 software (GraphPad Software).

### RNA sequencing

*Fn* strains SB010 and KCOM 3764 were grown on FAA plates (Oxoid, Thermo Fisher Scientific) supplemented with 10% DHB (Fisher Scientific). Plates were incubated at 37 °C in a Bactron600 anaerobic chamber (Sheldon Manufacturing) for 2 days. Subsequent lawns were prepared on FAA + 10% DHB plates and incubated at 37 °C in a Bactron600 anaerobic chamber for 2 days. Cells were resuspended in TSB (Becton Dickinson) and standardized to an optical density at 600 nm (OD_600nm_) of 0.5. The culture was split into triplicates for each condition and incubated under anaerobic conditions at 37 °C for 4 h. The conditions were as follows: TSB broth alone, TSB supplemented with 50 mM 1,2-PD (Fisher Scientific) and 20 nM vitamin B_12_ (Fisher Scientific) or TSB supplemented with 15 mM EA (Fisher Scientific) and 20 nM vitamin B_12_, for 4 h at 37 °C under anaerobic conditions. SB010 was further incubated in TSB supplemented with 20 nM vitamin B_12_ under the same conditions. Cells were pelleted at 8,000 r.p.m. for 5 min and washed once in 1× PBS and pelleted again under the same conditions. Cells were then washed once in RNAlater (Thermo Fisher) and pelleted again, and all supernatant was removed before storage at −80 °C. RNA was extracted using the RNeasy Extraction Kit (Qiagen) for Illumina Stranded RNA library preparation with RiboZero Plus rRNA depletion. RNA library was sequenced to a minimum read count of 12 million paired-end reads.

### Mouse model experiments

Multiple intestinal neoplasia (*Apc*^*Min+/−*^) mice were purchased (Jackson Laboratory, strain No. 002020). Female mice aged 6–8 weeks old were used for two experimental trials with three treatment arms each. Mice were randomly assigned to treatment arms. Mice were treated with streptomycin (2 mg ml^−1^; Sigma Aldrich) in drinking water for 7 days and then treated with 1.5% dextran sodium sulfate (MP Biomedical) in drinking water for 7 days to induce colitis and facilitate colonic tumours. Mice were then supplied with normal water for 24 h before receiving an oral gavage of *Fna* strains. Treatment arm 1 mice each received a 200 µl volume of PBS vehicle control, arm 2 mice each received 1 × 10^9^ *Fna* clade 1 (*Fna* C1) cells in a 200 µl volume, and arm 3 mice each received 1 × 10^9^ *Fna* clade 2 (*Fna* C2) cells in a 200 µl volume. The *Fna* C1 slurry was an equal mix of strains KCOM 3363, KCOM 3764 and SB048, and the *Fna* C2 slurry was an equal mix of strains SB001, SB010 and KCOM 2763. Strain mixes instead of single-strain representatives were chosen to capture a greater proportion of *Fna* clade-specific genes. *Fna* strains were grown on FAA plates (Oxoid, Thermo Fisher Scientific) supplemented with 10% DHB (Fisher Scientific). Plates were incubated at 37 °C in a Bactron600 anaerobic chamber (Sheldon Manufacturing) for 2–3 days. Subsequent lawns were prepared on FAA + 10% DHB plates and incubated at 37 °C in a Bactron600 anaerobic chamber for 2 days. For each *Fna* strain, cells were resuspended in PBS. Strain mixes were prepared by volume on the basis of OD_600nm_ standardized by each strain’s colony-forming units per millilitre at OD_600nm_ = 1 (*Fna* C1: KCOM 3363 6.71 × 10^7^, KCOM 3764 7.27 × 10^7^, SB048 1.97 × 10^8^; *Fna* C2: SB001 7.61 × 10^7^, SB010 5.00 × 10^8^, KCOM 2763 1.82 × 10^8^) for an equal mix of cells from each *Fna* C1 and each *Fna* C2 strain. Mice were monitored until the end-point (6 weeks post-gavage) when the mice were 15–17 weeks old. The Fred Hutchinson Cancer Center Animal Care and Use Committee approved all experimental protocols (IACUC PROTO202100004). All animal work complied with relevant ethical guidelines. Mice were housed on a 12-h light/12-h dark cycle with controlled temperature (65–75 °F (about 18–23 °C)) and humidity (40–60%). Maximal tumour size depended on the number of palpable tumours (1 tumour, maximum 2 cm diameter; 2 tumours, maximum 1.5 cm diameter; ≥3 tumours, maximum under veterinary discretion) and these limits were not exceeded. Intestinal sections from all mice (*n* = 8 per arm) were blindly assessed by pathology for intestinal adenoma load. To assess differences in intestinal adenoma load by treatment arm, *P* values were calculated by applying a one-way ANOVA using GraphPad Prism v.7.0 software (GraphPad Software).

### Intestinal metabolomics analysis

Metabolomic profiling was conducted using ultrahigh-performance liquid chromatography–tandem mass spectrometry by the metabolomics provider Metabolon on intestinal tissue sections from mice from the second mouse study (*n* = 4). The global discovery panel used by Metabolon includes 5,400+ metabolites in 70 major pathways, including metabolites of both eukaryotic and bacterial origin. Metabolic pathway enrichment analysis was carried out by Metabolon. Further analysis, including partial least squares discriminant analysis on detected metabolites and heat map clustering were carried out on sample-normalized data using MetaboAnalyst^70^, v.5.

### Mouse faecal DNA extraction and quantitative PCR

DNA was extracted from mouse faecal samples using the Zymo Quick-DNA Microprep Kit (Zymo Research) according to the manufacturer’s instructions. A custom TaqMan primer and probe set was used to amplify *Fusobacterium* genus DNA (Integrated DNA Technologies) as previously described^71^. The cycle threshold (Ct) values for the *Fusobacterium* genus were normalized to the input amount of mouse faecal genomic DNA in each reaction and were assayed in at least duplicate in 20-µl reactions containing 1× final concentration TaqMan Universal PCR Master Mix (Applied Biosystems) and the *Fusobacterium* TaqMan primer and probe, in a 96-well optical PCR plate. A positive control and non-template control were included in each quantitative PCR run. *Fusobacterium* copy numbers were estimated following the generation of a standard curve with pure *Fna* C1 and *Fna* C2 DNA input. Amplification and detection of DNA was carried out with the QuantStudio 3 Real-Time PCR System (Applied Biosystems) using the following reaction conditions: 10 min at 95 °C and 40 cycles of 15 s at 95 °C and 1 min at 60 °C. Ct was calculated using the automated settings (Applied Biosystems). The primer and probe sequences for the TaqMan assay are as follows: *Fusobacterium* genus forward primer, 5′-AAGCGCGTCTAGGTGGTTATGT-3′; *Fusobacterium* genus reverse primer, 5′-TGTAGTTCCGCTTACCTCTCCAG-3′; *Fusobacterium* genus FAM probe, 5′-CAACGCAATACAGAGTTGAGCCCTGCATT-3′.

### Biolog PM10 phenotype microarray plates

Biolog PM10 plates and corresponding IF-0a and IF-10b solutions were pre-reduced under anaerobic conditions at 4 °C overnight (AnaeroGen Gas Generating Systems, Oxoid, Thermo Fisher Scientific). *Fna* strains were grown on FAA plates (Oxoid, Thermo Fisher Scientific) supplemented with 10% DHB (Fisher Scientific). Plates were incubated at 37 °C in a Concept1000 anaerobic chamber (BakerRuskinn) for 24 h. Under these same anaerobic conditions, *Fna* cells were resuspended in 2 ml of pre-reduced IF-0a and normalized across all samples to OD_600nm_ = 0.179 as recommended by Biolog. The final suspension was prepared by combining 0.75 ml of normalized bacterial suspension with 11.25 ml of mix B (100 ml pre-reduced IF-10b with 1.2 ml dye mix D, and 11.18 ml pre-reduced sterile water) to a final volume of 12 ml. For each PM10 plate well, 100 μl of final suspension was added. The PM10 plate was then equilibrated to aerobic conditions at room temperature for 10 min, and then incubated under anaerobic, hydrogen-free conditions for 24 h at 37 °C (AnaeroGen Gas Generating Systems, Oxoid, Thermo Fisher Scientific). Plates were imaged and absorbance at 590 nm was quantified using a plate reader (Biotek).

### Glutaminase assay

*Fna* strains were grown on FAA plates (Oxoid, Thermo Fisher Scientific) supplemented with 10% DHB (Fisher Scientific) in a Concept1000 anaerobic chamber (BakerRuskinn) at 37 °C for 2 days. Sterile cotton swabs were used to resuspend cells in TSB (Becton Dickinson) supplemented with 2.5% yeast extract (Becton Dickinson) and 0.4 mg ml^−1^ l-cysteine (Alfa Aesar). *Fna* strains were grown in liquid culture in a Concept1000 anaerobic chamber (BakerRuskinn) at 37 °C for about 20 h. For each strain, 0.75 ml of culture standardized to OD_600nm_ = 1 was spun down at 7830 r.p.m. The cell pellet was resuspended in 1 ml of Gls solution. Gls solution contains 0.2 g l-glutamine (Sigma Aldrich), 0.01 g bromocresol green (Sigma Aldrich), 18 g sodium chloride (Sigma Aldrich), 0.6 ml Triton X-100 (Sigma Aldrich) and 200 ml deionized water. Gls solution is filter sterilized post pH adjustment to 3.1. For each strain, a 300 μl volume was aliquoted into a conical-bottom 96-well plate in triplicate and incubated anaerobically at 37 °C for 2 h. The plate was spun down for 1 min at 3,000 r.p.m. The supernatant was transferred to a flat-bottom 96-well plate and absorbance at 600 nm was quantified using a plate reader (Biotek).

### Acid resistance in simulated gastric fluid

*Fna* strains were grown on FAA plates (Oxoid, Thermo Fisher Scientific) supplemented with 10% DHB (Fisher Scientific) in a Concept1000 anaerobic chamber (BakerRuskinn) at 37 °C for 1–2 days. The cells were resuspended in 50 ml TSB (Becton Dickinson) supplemented with 2.5% yeast extract (Becton Dickinson) and 0.4 mg ml^−1^ l-cysteine (Alfa Aesar). The cells were grown in liquid culture in a Concept1000 anaerobic chamber (BakerRuskinn) at 37 °C for 25 h. All strains were standardized to an OD_600nm_ = 1 in 5 ml of supplemented TSB, simulated gastric fluid (Biochemazone) at pH 3 or simulated gastric fluid supplemented with 10 mM glutamate (Sigma Aldrich) at pH 3. Every 10 min, 10 μl of each suspension was spotted on FAA + 10% DHB plates. Plates were incubated anaerobically in a Concept1000 anaerobic chamber (BakerRuskinn) at 37 °C for 3 days.

### Patient specimens

All patient tumour tissue included in the analysis was diagnosed colorectal adenocarcinoma. For patient cohort 1, patients signed an informed consent for the collection and analysis of their tumour specimens. The use of patient specimens for this work was approved by the Fred Hutchinson Cancer Center Institutional Review Board under protocol numbers RG 1006552, 1005305, 1006664 and 1006974. Patient age, sex and ethnicity were not selection criteria for specimen acquisition. For microbial culturing efforts, primary CRC tumours that were treatment naive were prioritized. For patient cohort 2, samples from BioProject PRJNA362951 were used.

### Bacterial 16S rRNA gene sequencing

DNA was extracted from patient tissue as described previously^6^ and processed with the ZymoBIOMICS Service - Targeted Metagenomic Sequencing (Zymo Research). Bacterial V3–V4 16S ribosomal RNA gene-targeted sequencing was carried out. The V3–V4 targeting primers have been custom-designed by Zymo Research to provide the best coverage of the 16S gene while maintaining high sensitivity. They are based on the general bacterial 16S rRNA gene primers 341F (CCTACGGGNGGCWGCAG) and 805R (GACTACHVGGGTATCTAATCC), which amplify the V3–V4 region of the 16S rRNA gene. The amplification was carried out at a higher annealing temperature to ensure only bacterial sequences were amplified. An extraction control was included and showed no amplification during the library preparation (run to 42 cycles). The sequencing library was prepared using the AccuBIOME Amplicon Sequencing Kit (Zymo Research), in which PCR reactions were carried out in real-time PCR machines to prevent PCR chimera formation. The amplicon libraries were cleaned up with Zymo Research’s Select-a-Size DNA Clean & Concentrator (>200-base-pair fragments were kept), quantified with TapeStation, normalized and pooled together. The final library was quantified with quantitative PCR and sequenced on an Illumina MiSeq with a v3 reagent kit (600 cycles). The sequencing was carried out with >10% PhiX mix and in paired-end mode. Raw sequence reads were trimmed with Trimmomatic-0.33 (ref. ^72^). *Fna* clade-specific amplicon sequence variants were designed by the provider CosmosID. We provided 16S rRNA gene sequences for all *Fna* C1 and *Fna* C2 strains. As the 16S sequence of *Fna* C1 branched closely with *Fnv* (Extended Data Fig. 2a), we additionally provided the 16S rRNA gene sequences for all *Fnv* strains, to ensure the specificity of an *Fna* C1 amplicon sequence variant that would not detect *Fnv*. A custom SILVA database was generated using these 16S rRNA gene sequences and SILVA 138.1 SSU Ref. NR99 version, and the DADA2 version of the species training set. First, all sequences in the SILVA database that matched with supplied sequences were removed from SILVA. Next, the custom sequences were added into the SILVA database file, in which the species names were appended on the basis of supplied metadata info (*Fna* C1, *Fna* C2 or *Fnv*). Analysis on this database was then run through the nf-core AmpliSeq pipeline, with the parameters --FW_primer CCTACGGGRSGCAGCA, --RV_primer GACTACHVGGGTATCT, --trunc_qmin 20, --trunc_rmin 0.2, --max_ee 6, --min-frequency 1, --picrust, and -- dada_ref_tax_custom.

### Meta-analysis of *Fna* clades in relation to CRC using publicly available shotgun metagenomic samples

To study the association between each *Fna* clade and CRC, we profiled shotgun stool metagenomic samples from 9 publicly available cohorts (Supplementary Table 22), for a total of 627 patients with CRC and 619 healthy individuals using MetaPhlAn4 (ref. ^63^; https://github.com/biobakery/biobakery/wiki/metaphlan4) against an *Fna* clade-specific database generated from our *Fna* genomes, which are available at the National Centre for Biotechnology Information (NCBI) under the BioProject accession number PRJNA549513. A distinct species-level genome bin (SGB)^73^ could be identified for each *Fn* subspecies and *Fna* clade (*Fna* C1: SGB6013, *Fna* C2: SGB6007, *Fnn*: SGB6011, *Fnp*: SGB6001, *Fnv*: SGB6014). Each SGB was associated with the sample condition fitting an ordinary least squares model of the shape: arcsin-squared-root-transformed SGB abundance ~ study condition + *C*(sex) + age + BMI + sequencing depth of sample. For each model, an adjusted standardized mean difference between the two study conditions was extracted as previously described^74^: standardized mean difference = (*t × *(*n*1 + *n*2))/(sqrt(*n*1 + *n*2) × sqrt(*n*1 + *n*2 − 2)), in which *t* defines the *t*-score of the corresponding variable, *n*1 is the number of samples in the zero class, *n*2 is the number of samples in the one class, and *n*1 + *n*2 − 2 are the degrees of freedom for the model. Corresponding standard errors were computed as: s.e. = sqrt(((*n*1 + *n*2 − 1)/(*n*1 + *n*2 −3)) × (4/(*n*1 + *n*2)) × (1 + (((standardized mean difference)^2^)/8))). Statistical significance was assessed by the two-tailed Wald test. Effect sizes were pooled and analysed using random-effect meta-analysis^75^ using the Paule–Mandel heterogeneity estimator^76^. The statistical significance of the meta-analysis was computed as the *z*-score of the null hypothesis that the average effect is zero^75^. All *P* values were corrected using the Benjamini–Yakuteli method.

### Mapping of putative *eut*, *pdu* and *gdar* operons in publicly available metagenomic samples

To assess the presence of putative *eut*, *pdu* and *gdar* system operons in patients with CRC compared to healthy individuals, we profiled shotgun stool metagenomic samples from 9 publicly available cohorts (Supplementary Table 22), for a total of 627 patients with CRC and 619 healthy individuals. Metagenomic samples were mapped against the *Fna* SB010 *eut*, *pdu* and *gdar* operons using Bowtie2 (version 2.4.5, --sensitive parameter)^77^. Breadth and depth of coverage of each gene in the operons was assessed using the breadth_depth.py script of the CMSeq tool (parameters --minqual 30 --mincov 1)^78^. Detected genes had a breadth of coverage threshold above 50%. For *eut* and *pdu* results, putative operons had a threshold of presence of 90% of *eut* and *pdu* genes relative to the *Fna* SB010 operon structures. For *gdar* results, putative operons had a threshold of presence of 100% of *gdar* genes relative to the *Fna* SB010 operon structure.

### Reporting summary

Further information on research design is available in the Nature Portfolio Reporting Summary linked to this article.

## Online content

Any methods, additional references, Nature Portfolio reporting summaries, source data, extended data, supplementary information, acknowledgements, peer review information; details of author contributions and competing interests; and statements of data and code availability are available at 10.1038/s41586-024-07182-w.

### Supplementary information

Supplementary Information Legends for Supplementary Tables 1–25.

Reporting Summary

Supplementary Tables Supplementary Tables 1–25.

### Source data

Source Data Fig. 4

Source Data Extended Data Fig. 6

## Data Availability

All genomes from this study are available at the NCBI under the BioProject accession number PRJNA549513 and all methylomes are available in the Restriction Enzyme Database (REBASE). Raw sequencing data from RNA-sequencing experiments are available in the NCBI Sequence Read Archive repository under the BioProject accession number PRJNA937266. Raw sequencing data from 16S rRNA sequencing experiments are available in the NCBI Sequence Read Archive repository under the BioProject accession number PRJNA1064180. Source data are provided with this paper.

## References

[1] Segata N (2012). Composition of the adult digestive tract bacterial microbiome based on seven mouth surfaces, tonsils, throat and stool samples. Genome Biol..

[2] Kostic AD (2012). Genomic analysis identifies association of *Fusobacterium* with colorectal carcinoma. Genome Res..

[3] Kostic AD (2013). *Fusobacterium nucleatum* potentiates intestinal tumorigenesis and modulates the tumor-immune microenvironment. Cell Host Microbe.

[4] Flanagan L (2014). *Fusobacterium nucleatum* associates with stages of colorectal neoplasia development, colorectal cancer and disease outcome. Eur. J. Clin. Microbiol. Infect. Dis..

[5] Mima K (2016). *Fusobacterium nucleatum* in colorectal carcinoma tissue and patient prognosis. Gut.

[6] Bullman S (2017). Analysis of *Fusobacterium* persistence and antibiotic response in colorectal cancer. Science.

[7] Yu T (2017). *Fusobacterium nucleatum* promotes chemoresistance to colorectal cancer by modulating autophagy. Cell.

[8] Serna G (2020). *Fusobacterium nucleatum* persistence and risk of recurrence after preoperative treatment in locally advanced rectal cancer. Ann. Oncol..

[9] LaCourse, K. D., Johnston, C. D. & Bullman, S. The relationship between gastrointestinal cancers and the microbiota. *Lancet Gastroenterol. Hepatol*. **6**, 498–509 (2021). 10.1016/S2468-1253(20)30362-9 PMC10773981 33743198

[10] Galeano Niño JL (2022). Effect of the intratumoral microbiota on spatial and cellular heterogeneity in cancer. Nature.

[11] LaCourse KD (2022). The cancer chemotherapeutic 5-fluorouracil is a potent *Fusobacterium nucleatum* inhibitor and its activity is modified by intratumoral microbiota. Cell Rep..

[12] Allen-Vercoe E, Strauss J, Chadee K (2011). *Fusobacterium nucleatum*: an emerging gut pathogen?. Gut Microbes.

[13] Manson McGuire A (2014). Evolution of invasion in a diverse set of *Fusobacterium* species. mBio.

[14] Holt RA, Cochrane K (2017). Tumor potentiating mechanisms of *Fusobacterium nucleatum*, a multifaceted microbe. Gastroenterology.

[15] Ponath F, Zhu Y, Cosi V, Vogel J (2022). Expanding the genetic toolkit helps dissect a global stress response in the early-branching species *Fusobacterium nucleatum*. Proc. Natl Acad. Sci. USA.

[16] Queen J (2022). Comparative analysis of colon cancer-derived *Fusobacterium nucleatum* subspecies: inflammation and colon tumorigenesis in murine models. mBio.

[17] Tomkovich S (2017). Locoregional effects of microbiota in a preclinical model of colon carcinogenesis. Cancer Res..

[18] Eid J (2009). Real-time DNA sequencing from single polymerase molecules. Science.

[19] Komiya Y (2019). Patients with colorectal cancer have identical strains of *Fusobacterium nucleatum* in their colorectal cancer and oral cavity. Gut.

[20] Abed J (2020). Colon cancer-associated *Fusobacterium nucleatum* may originate from the oral cavity and reach colon tumors via the circulatory system. Front. Cell. Infect. Microbiol..

[21] Eren AM (2015). Anvi’o: an advanced analysis and visualization platform for ‘omics data. PeerJ.

[22] Tettelin, H. et al. Genome analysis of multiple pathogenic isolates of *Streptococcus agalactiae*: implications for the microbial “pan-genome”. *Proc. Natl Acad. Sci. USA* **102** 13950–13955 (2005). 10.1073/pnas.0506758102 PMC1216834 16172379

[23] Tettelin, H. & Medini, D. (eds) *The Pangenome: Diversity, Dynamics and Evolution of Genomes* (Springer, 2020). 32633908

[24] Ang MY (2016). Comparative genome analysis of *Fusobacterium nucleatum*. Genome Biol. Evol..

[25] Horesh G (2021). Different evolutionary trends form the twilight zone of the bacterial pan-genome. Microb. Genomics.

[26] Shaiber A (2020). Functional and genetic markers of niche partitioning among enigmatic members of the human oral microbiome. Genome Biol..

[27] Aramaki T (2020). KofamKOALA: KEGG ortholog assignment based on profile HMM and adaptive score threshold. Bioinformatics.

[28] Borozan I (2022). Molecular and pathology features of colorectal tumors and patient outcomes are associated with *Fusobacterium nucleatum* and its subspecies *animalis*. Cancer Epidemiol. Biomarkers Prev..

[29] Strauss J (2011). Invasive potential of gut mucosa-derived *Fusobacterium nucleatum* positively correlates with IBD status of the host. Inflamm. Bowel Dis..

[30] Kook J-K (2017). Genome-based reclassification of *Fusobacterium nucleatum* subspecies at the species level. Curr. Microbiol..

[31] Abed J (2016). Fap2 mediates *Fusobacterium nucleatum* colorectal adenocarcinoma enrichment by binding to tumor-expressed Gal-GalNAc. Cell Host Microbe.

[32] Bachrach G, Rosen G, Bellalou M, Naor R, Sela MN (2004). Identification of a *Fusobacterium nucleatum* 65 kDa serine protease. Oral Microbiol. Immunol..

[33] Casasanta MA (2017). A chemical and biological toolbox for Type Vd secretion: characterization of the phospholipase A1 autotransporter FplA from *Fusobacterium nucleatum*. J. Biol. Chem..

[34] Coppenhagen-Glazer S (2015). Fap2 of *Fusobacterium nucleatum* is a galactose-inhibitable adhesin involved in coaggregation, cell adhesion, and preterm birth. Infect. Immun..

[35] Kaplan CW (2005). *Fusobacterium nucleatum* apoptosis-inducing outer membrane protein. J. Dent. Res..

[36] Kaplan CW, Lux R, Haake SK, Shi W (2009). The *Fusobacterium nucleatum* outer membrane protein RadD is an arginine-inhibitable adhesin required for inter-species adherence and the structured architecture of multispecies biofilm. Mol. Microbiol..

[37] Lima BP, Shi W, Lux R (2017). Identification and characterization of a novel *Fusobacterium nucleatum* adhesin involved in physical interaction and biofilm formation with *Streptococcus gordonii*. MicrobiologyOpen.

[38] Han YW (2005). Identification and characterization of a novel adhesin unique to oral fusobacteria. J. Bacteriol..

[39] Xu M (2007). FadA from *Fusobacterium nucleatum* utilizes both secreted and nonsecreted forms for functional oligomerization for attachment and invasion of host cells. J. Biol. Chem..

[40] Fardini Y (2011). *Fusobacterium nucleatum* adhesin FadA binds vascular endothelial cadherin and alters endothelial integrity: VE-cadherin is a novel receptor for *F. nucleatum*. Mol. Microbiol..

[41] Rubinstein MR (2013). *Fusobacterium nucleatum* promotes colorectal carcinogenesis by modulating E-cadherin/β-catenin signaling via its FadA adhesin. Cell Host Microbe.

[42] Umaña A (2019). Utilizing whole *Fusobacterium* genomes to identify, correct, and characterize potential virulence protein families. J. Bacteriol..

[43] Gursoy UK, Pöllänen M, Könönen E, Uitto V-J (2010). Biofilm formation enhances the oxygen tolerance and invasiveness of *Fusobacterium nucleatum* in an oral mucosa culture model. J. Periodontol..

[44] Kim H-S (2010). Application of rpoB and zinc protease gene for use in molecular discrimination of *Fusobacterium nucleatum* subspecies. J. Clin. Microbiol..

[45] Gardner SN, Slezak T, Hall BG (2015). kSNP3.0: SNP detection and phylogenetic analysis of genomes without genome alignment or reference genome. Bioinformatics.

[46] Richter M, Rosselló-Móra R (2009). Shifting the genomic gold standard for the prokaryotic species definition. Proc. Natl Acad. Sci. USA.

[47] Russo E (2023). From adenoma to CRC stages: the oral-gut microbiome axis as a source of potential microbial and metabolic biomarkers of malignancy. Neoplasia.

[48] Callahan BJ, McMurdie PJ, Holmes SP (2017). Exact sequence variants should replace operational taxonomic units in marker-gene data analysis. ISME J..

[49] Gur C (2015). Binding of the Fap2 protein of *Fusobacterium nucleatum* to human inhibitory receptor TIGIT protects tumors from immune cell attack. Immunity.

[50] Yang DC, Blair KM, Salama NR (2016). Staying in shape: the impact of cell shape on bacterial survival in diverse environments. Microbiol. Mol. Biol. Rev..

[51] Gautreau G (2020). PPanGGOLiN: depicting microbial diversity via a partitioned pangenome graph. PLoS Comput. Biol..

[52] Pacheco AR, Sperandio V (2015). Enteric pathogens exploit the microbiota-generated nutritional environment of the gut. Microbiol. Spectr..

[53] Biase DD, Pennacchietti E (2012). Glutamate decarboxylase‐dependent acid resistance in orally acquired bacteria: function, distribution and biomedical implications of the gadBC operon. Mol. Microbiol..

[54] Tanaka T (2006). Dextran sodium sulfate strongly promotes colorectal carcinogenesis in *Apc*^*Min/+*^ mice: inflammatory stimuli by dextran sodium sulfate results in development of multiple colonic neoplasms. Int. J. Cancer.

[55] Kennedy L, Sandhu JK, Harper M-E, Cuperlovic-Culf M (2020). Role of glutathione in cancer: from mechanisms to therapies. Biomolecules.

[56] Pompella A, De Tata V, Paolicchi A, Zunino F (2006). Expression of γ-glutamyltransferase in cancer cells and its significance in drug resistance. Biochem. Pharmacol..

[57] Hanigan, M. H. Gamma-glutamyl transpeptidase. *Adv. Cancer Res.* **122**, 103–141 (2014). 10.1016/B978-0-12-420117-0.00003-7 PMC4388159 24974180

[58] Rao JN, Xiao L, Wang J-Y (2020). Polyamines in gut epithelial renewal and barrier function. Physiology.

[59] Morad SAF, Cabot MC (2013). Ceramide-orchestrated signalling in cancer cells. Nat. Rev. Cancer.

[60] Castellarin M (2012). *Fusobacterium nucleatum* infection is prevalent in human colorectal carcinoma. Genome Res..

[61] Dejea CM (2018). Patients with familial adenomatous polyposis harbor colonic biofilms containing tumorigenic bacteria. Science.

[62] Engevik MA (2021). *Fusobacterium nucleatum* adheres to *Clostridioides difficile* via the RadD adhesin to enhance biofilm formation in intestinal mucus. Gastroenterology.

[63] Blanco-Miguez, A. et al. Extending and improving metagenomic taxonomic profiling with uncharacterized species with MetaPhlAn 4. *Nat. Biotechnol*. **41**, 1633–1644 (2023). 10.1038/s41587-023-01688-w PMC10635831 36823356

[64] Chaumeil P-A, Mussig AJ, Hugenholtz P, Parks DH (2020). GTDB-Tk: a toolkit to classify genomes with the Genome Taxonomy Database. Bioinformatics.

[65] Kumar S, Stecher G, Li M, Knyaz C, Tamura K (2018). MEGA X: molecular evolutionary genetics analysis across computing platforms. Mol. Biol. Evol..

[66] Letunic I, Bork P (2021). Interactive Tree Of Life (iTOL) v5: an online tool for phylogenetic tree display and annotation. Nucleic Acids Res..

[67] Payne LJ (2021). Identification and classification of antiviral defence systems in bacteria and archaea with PADLOC reveals new system types. Nucleic Acids Res..

[68] Zhou Y, Liang Y, Lynch KH, Dennis JJ, Wishart DS (2011). PHAST: a fast phage search tool. Nucleic Acids Res..

[69] Arndt D (2016). PHASTER: a better, faster version of the PHAST phage search tool. Nucleic Acids Res..

[70] Xia J, Psychogios N, Young N, Wishart DS (2009). MetaboAnalyst: a web server for metabolomic data analysis and interpretation. Nucleic Acids Res..

[71] Martin FE, Nadkarni MA, Jacques NA, Hunter N (2002). Quantitative microbiological study of human carious dentine by culture and real-time PCR: association of anaerobes with histopathological changes in chronic pulpitis. J. Clin. Microbiol..

[72] Bolger AM, Lohse M, Usadel B (2014). Trimmomatic: a flexible trimmer for Illumina sequence data. Bioinformatics.

[73] Pasolli E (2019). Extensive unexplored human microbiome diversity revealed by over 150,000 genomes from metagenomes spanning age, geography, and lifestyle. Cell.

[74] Nakagawa S, Cuthill IC (2007). Effect size, confidence interval and statistical significance: a practical guide for biologists. Biol. Rev..

[75] Borenstein, M., Hedges, L. V., Higgins, J. P. T & Rothstein H. R. *Introduction to Meta-Analysis* (Wiley, 2021).

[76] Veroniki AA (2016). Methods to estimate the between‐study variance and its uncertainty in meta‐analysis. Res. Synth. Methods.

[77] Langmead B, Salzberg SL (2012). Fast gapped-read alignment with Bowtie 2. Nat. Methods.

[78] Zolfo M (2019). Detecting contamination in viromes using ViromeQC. Nat. Biotechnol..

