# Supplementary material for: A distinct Fusobacterium nucleatum clade dominates the colorectal cancer niche
Source: Nature. 2024 Mar 20;628(8007):424–32. doi: 10.1038/s41586-024-07182-w (PMC11006615; doi:10.1038/s41586-024-07182-w)
Supplement: Supplementary file 1 — Legends for Supplementary Tables 1–25. [file 41586_2024_7182_MOESM1_ESM.pdf]

nature portfolio

https://doi.org/10.1038/s41586-024-07182-w

Supplementary information

A distinct *Fusobacterium nucleatum* clade dominates the colorectal cancer niche

In the format provided by the authors and unedited

nature portfolio

https://doi.org/10.1038/s41586-024-07182-w

Supplementary information

A distinct *Fusobacterium nucleatum* clade dominates the colorectal cancer niche

In the format provided by the authors and unedited

Supplementary Information Guide

Table of Contents

- Supplementary Table Legends: pages 2-5

Supplementary File contains:

- Supplementary Tables 1-25

Supplementary Table Legends

Supplementary Table 1: Strain Data Table
Table indicates each bacterial strain and its niche of origin sequenced for this study. The corresponding genome biosample and accession numbers as well as the methylome REBASE[1] identifier are shown. For each genome, its size, extrachromosomal elements, and phylogenetic classification (see Methods) are indicated.

Supplementary Table 2: GTDB-Tk phylogenetic classification for our genome collection
For each Fusobacterium nucleatum genome in our collection table indicates the assigned phylogenetic classification as determined by GTDB-Tk[2].

Supplementary Table 3: *Fusobacterium nucleatum* Anvi'o pangenomic analysis
The *Fusobacterium nucleatum* pangenome of colorectal cancer-associated and oral-associated strains was characterized using the analysis and visualization platform for microbial 'omics (Anvi'o)[3]. Table indicates each gene cluster (GC) identified and its corresponding niche-enrichment association, enrichment score, and q-value. GCs were classified into a pangenome partition based on relative presence (Fig. 1c). KEGG ortholog mapping via KofamKOALA[4] was used to match each GC to a KO identifier for its putative function (Fig. 1e).

Supplementary Table 4: Average nucleotide identity (ANI) ranges between *Fusobacterium nucleatum* subspecies and *F. nucleatum* subspecies *animalis* clades
Table shows the range of average nucleotide identity (ANI) scores between each *Fusobacterium nucleatum* (*Fn*) subspecies and each *Fna* clade as derived by Anvi'o[3] from Supplementary Table 5. Colors indicate phylogenetic assignment by *Fn* subspecies *animalis* (*Fna*) clade (*Fna* C1 green, *Fna* C2 lavender) and *Fn* subspecies (*Fnn* gold, *Fnp* purple, *Fnv* brown).

Supplementary Table 5: Average nucleotide identity (ANI) scores between *Fusobacterium nucleatum* genomes
Table shows the individual average nucleotide identity (ANI) scores between each *Fusobacterium nucleatum* (*Fn*) genome pair as derived by Anvi'o[3]. Colors indicate phylogenetic assignment by *Fna* clade (*Fna* C1 green, *Fna* C2 lavender) and *Fn* subspecies (*Fnn* gold, *Fnp* purple, *Fnv* brown).

Supplementary Table 6: *Fusobacterium nucleatum* plasmid annotations
Of the 135 *Fusobacterium nucleatum* strains sequenced, 25 strains harbored 41 putative plasmids (extrachromosomal elements). Table indicates the originating strain for each plasmid, its size, and its annotation as determined by both PlasMapper[5] and pLannotate[6].

Supplementary Table 7: Methyl-modified motifs across *Fusobacterium nucleatum* genomes
Each *Fusobacterium nucleatum* strain's methyl-modified nucleotides were analyzed using Single-Molecule Real Time Sequencing (SMRTSeq)[7] kinetics (Basemod analysis) and the predicted methyl-modified nucleotide motif acquired via REBASE[1] analysis. Table indicates the presence (1) versus absence (0) of each methyl-modified motif across *Fn* genomes, ordered by *Fna* clade (*Fna* C1 green, *Fna* C2 lavender) and *Fn* subspecies (*Fnn* gold, *Fnp* purple, *Fnv* brown). The proportion of genomes within each phylogenetic grouping in which the methyl-modified motif was detected is indicated.

Supplementary Table 8: Amplicon sequence variants to distinguish *Fna* clades
Table notes amplicon sequence variant[8] sequences detected by CosmosID to distinguish *Fna* C1 and *Fna* C2 abundance in patient tissue specimens.

Supplementary Table 9: *Fusobacterium nucleatum* subspecies *animalis* Anvi'o pangenomic analysis with clade-based functional enrichment
The *Fusobacterium nucleatum* subspecies *animalis* pangenome was characterized using the analysis and visualization platform for microbial 'omics (Anvi'o)[3]. Table indicates each gene cluster (GC) identified and its corresponding *Fna* clade-enrichment association, enrichment score, and q-value. GCs were classified into a pangenome partition based on relative presence (Extended Data Fig. 2d). KEGG ortholog mapping via KofamKOALA[4] was used to match each GC to a KO identifier for its putative function (Extended Data Fig. 3e).

Supplementary Table 10: *Fusobacterium nucleatum* subspecies *animalis* PPanGGOLiN pangenomic analysis
The *Fusobacterium nucleatum* subspecies *animalis* (*Fna*) pangenome was characterized using the partitioned pangenome graph of linked neighboors (PPanGGOLiN) tool[9]. Table indicates each gene node (gene group) identified across *Fna* genomes, their module assignment, and annotated functions. The proportion of each node across *Fna* clades is noted. A PPanGGOLiN alignment was used to map each *Fna* PPanGGOLiN node to corresponding *Fna* Anvi'o gene clusters (Supplementary Table 9) and results are noted.

Supplementary Table 11: RNA-Sequencing of KCOM 3764 exposed to ethanolamine and Vitamin B12
Table notes results from RNA-Sequencing (see Methods) of KCOM 3764, a representative *Fna* C1 strain, exposed to 15mM of ethanolamine and 20nM of vitamin B12 for 4 hours (Extended Data Fig. 4b). PPanGGOLiN[9] node information for each KCOM 3764 gene is indicated. Statistical analysis performed using glmQLFTest, 2-sided.

Supplementary Table 12: RNA-Sequencing of KCOM 3764 exposed to 1,2-propanediol and Vitamin B12
Table notes results from RNA-Sequencing (see Methods) of KCOM 3764, a representative *Fna* C1 strain, exposed to 50mM of 1,2-propanediol and 20nM of vitamin B12 for 4 hours (Extended Data Fig. 4c). PPanGGOLiN[9] node information for each KCOM 3764 gene is indicated. Statistical analysis performed using glmQLFTest, 2-sided.

Supplementary Table 13: RNA-Sequencing of SB010 exposed to ethanolamine and Vitamin B12
Table notes results from RNA-Sequencing (see Methods) of SB010, a representative *Fna* C2 strain, exposed to 15mM of ethanolamine and 20nM of vitamin B12 for 4 hours (Extended Data Fig. 4b). PPanGGOLiN[9] node information for each SB010 gene is indicated. Mapping of KCOM 3764 and SB010 genes to a common PPanGGOLiN node allowed a comparison of their transcriptional responses. Genes that were significantly upregulated (with log2-transformed fold change ≥ 0.58 and -log10(p-value) ≥ 1.30) or downregulated (with log2-transformed fold change ≤ -0.58 and -log10(p-value) ≥ 1.30) in both KCOM 3764 and SB010 were removed to identify genes uniquely

differentially regulated in SB010 upon ethanolamine and Vitamin B12 exposure (Fig. 3e). Statistical analysis performed using glmQLFTest, 2-sided.

Supplementary Table 14: RNA-Sequencing of SB010 exposed to 1,2-propanediol and Vitamin B12

Table notes results from RNA-Sequencing (see Methods) of SB010, a representative *Fna* C2 strain, exposed to 50mM of 1,2-propanediol and 20nM of vitamin B12 for 4 hours (Extended Data Fig. 4c). PPanGGOLiN[9] node information for each SB010 gene is indicated. Mapping of KCOM 3764 and SB010 genes to a common PPanGGOLiN node allowed a comparison of their transcriptional responses. Genes that were significantly upregulated (with log2-transformed fold change ≥ 0.58 and -log10(p-value) ≥ 1.30) or downregulated (with log2-transformed fold change ≤ -0.58 and -log10(p-value) ≥ 1.30) in both KCOM 3764 and SB010 were removed to identify genes uniquely differentially regulated in SB010 upon 1,2-propanediol and Vitamin B12 exposure (Fig. 3f). Statistical analysis performed using glmQLFTest, 2-sided.

Supplementary Table 15: RNA-Sequencing of SB010 exposed to Vitamin B12

Table notes results from RNA-Sequencing (see Methods) of SB010, a representative *Fna* C2 strain, exposed to 20nM of vitamin B12 for 4 hours (Extended Data Fig. 4d). PPanGGOLiN[9] node information for each SB010 gene is indicated. Statistical analysis performed using glmQLFTest, 2-sided.

Supplementary Table 16: Metabolon metabolite analysis of Apc$^{Min+/-}$ mice intestinal tissue

Apc$^{Min+/-}$ mice, a murine model of colorectal cancer[10], were orally gavaged with either vehicle control (Arm 1), or representative strains of *Fna* C1 (Arm 2) and *Fna* C2 (Arm 3). Table notes results and comparisons of LC-MS global metabolomics on intestinal tissue from each treatment arm. Statistical analysis performed using glmQLFTest, 2-sided.

Supplementary Table 17: Metabolic pathway enrichment scores for Apc$^{Min+/-}$ mice intestinal tissue

Apc$^{Min+/-}$ mice, a murine model of colorectal cancer[10], were orally gavaged with either vehicle control (Arm 1), or representative strains of *Fna* C1 (Arm 2) and *Fna* C2 (Arm 3). Table notes metabolic pathway enrichment scores (see Methods) for each pairwise treatment comparison. Statistical analysis performed using glmQLFTest, 2-sided.

Supplementary Table 18: Details on patient samples (Cohort 1) for 16S microbiome analysis

Table notes patient ID, tissue type, 16S read identifiers and SRA accessions for 116 resected tumor tissue patient samples from treatment-naïve patients with colorectal cancer and for adjacent normal tissue from 62 of these patients.

Supplementary Table 19: Microbial relative abundance in paired normal adjacent and CRC tumor tissue specimens from CRC Cohort 1

Table notes relative abundance by 16S rRNA gene sequencing on resected tumor tissue from 62 patients with treatment naive colorectal cancer and on adjacent normal tissue (Fig. 5a-b).

Supplementary Table 20: Microbial relative abundance in primary tumor tissue specimens from CRC Cohort 1

Table notes relative abundance by 16S rRNA gene sequencing on resected tumor tissue from 116 patients with treatment naive colorectal cancer (Fig. 5a-b, Supplementary Table 18). Of these, the data for the 62 tumor specimens with a corresponding normal adjacent specimen are also included in Supplementary Table 19.

Supplementary Table 21: Microbial relative abundance in primary tumor tissue specimens from CRC Cohort 2
Table notes relative abundance by 16S rRNA gene sequencing on tumor tissue from 86 patients with colorectal cancer[11] (Fig. 5b).

Supplementary Table 22: Details on metagenomic datasets from nine independent cohorts used for meta-analysis
For each of the nine cohorts used in the meta-analysis, table notes the cohort's geographic origin, sequencing approach, and sequencing technology implemented.

Supplementary Table 23: *Fna* relative abundance in stool sample metagenomes from nine independent cohorts of patients with CRC and healthy controls
Table notes the relative abundance of *Fna* C1 and *Fna* C2 from stool sample metagenomes from 627 patients with colorectal cancer and 619 healthy individuals (Fig. 5c, Extended Data Fig. 10).

Supplementary Table 24: Meta-analysis of stool sample metagenomes from nine independent cohorts of patients with CRC and healthy controls
Table notes the results of a meta-analysis of standardized mean differences by random effects model from stool sample metagenomes from nine independent cohorts of patients with colorectal cancer and healthy controls (Extended Data Fig. 11). For each study, the the effect, standard error (SE), p-value, and q-value are reported. A pooled analysis including all 627 samples from patients with CRC and 619 healthy controls is also indicated ("RE"). Statistical significance assessed using Wald test, two-sided and p-value corrected via Benjamini-Yakuteli method.

Supplementary Table 25: Meta-analysis of stool sample metagenomes excluding *Fna* C1 and *Fna* C2 co-occurrence from nine independent cohorts of patients with CRC and healthy controls
Table notes the results of a meta-analysis of standardized mean differences by random effects model from stool sample metagenomes from nine independent cohorts of patients with colorectal cancer and healthy controls (Extended Data Fig. 11). Samples where *Fna* C1 and *Fna* C2 co-occurred were excluded. For each study, the the effect, standard error (SE), p-value, and q-value are reported. A pooled analysis including all 596 samples from patients with CRC and 616 healthy controls is also indicated ("RE"). Statistical significance assessed using Wald test, two-sided and p-value corrected via Benjamini-Yakuteli method.

References

1. Roberts, R. J., Vincze, T., Posfai, J. & Macelis, D. REBASE—a database for DNA restriction and modification: enzymes, genes and genomes. *Nucleic Acids Res.* **43**, D298–D299 (2015).
2. Chaumeil, P.-A., Mussig, A. J., Hugenholtz, P. & Parks, D. H. GTDB-Tk: a toolkit to classify genomes with the Genome Taxonomy Database. *Bioinformatics* **36**, 1925–1927 (2020).
3. Eren, A. M. *et al.* Anvi'o: an advanced analysis and visualization platform for 'omics data. *PeerJ* **3**, e1319 (2015).
4. Aramaki, T. *et al.* KofamKOALA: KEGG Ortholog assignment based on profile HMM and adaptive score threshold. *Bioinformatics* **36**, 2251–2252 (2020).
5. Dong, X., Stothard, P., Forsythe, I. J. & Wishart, D. S. PlasMapper: a web server for drawing and auto-annotating plasmid maps. *Nucleic Acids Res.* **32**, W660–W664 (2004).
6. McGuffie, M. J. & Barrick, J. E. pLannotate: engineered plasmid annotation. *Nucleic Acids Res.* **49**, W516–W522 (2021).
7. Eid, J. *et al.* Real-Time DNA Sequencing from Single Polymerase Molecules. *Sci. 2009 Jan 23235910133-8* **323**, 7 (2009).
8. Callahan, B. J., McMurdie, P. J. & Holmes, S. P. Exact sequence variants should replace operational taxonomic units in marker-gene data analysis. *ISME J.* **11**, 2639–2643 (2017).
9. Gautreau, G. *et al.* PPanGGOLiN: Depicting microbial diversity via a partitioned pangenome graph. *PLOS Comput. Biol.* **16**, e1007732 (2020).
10. Tanaka, T. *et al.* Dextran sodium sulfate strongly promotes colorectal carcinogenesis in *Apc* $^{Min/+}$ mice: Inflammatory stimuli by dextran sodium sulfate results in development of multiple colonic neoplasms. *Int. J. Cancer* **118**, 25–34 (2006).

11. Bullman, S. *et al.* Analysis of *Fusobacterium* persistence and antibiotic response in colorectal cancer. *Science* **358**, 1443–1448 (2017).
