# Supplementary material for: A distinct Fusobacterium nucleatum clade dominates the colorectal cancer niche
Source: Nature. 2024 Mar 20;628(8007):424–32. doi: 10.1038/s41586-024-07182-w (PMC11006615; doi:10.1038/s41586-024-07182-w)
Supplement: Supplementary file 2 — Reporting Summary [file 41586_2024_7182_MOESM2_ESM.pdf]

nature portfolio

Corresponding author(s): Susan Bullman
Christopher Johnston

Last updated by author(s): November 14, 2023

Reporting Summary

Nature Portfolio wishes to improve the reproducibility of the work that we publish. This form provides structure for consistency and transparency in reporting. For further information on Nature Portfolio policies, see our Editorial Policies and the Editorial Policy Checklist.

Statistics

For all statistical analyses, confirm that the following items are present in the figure legend, table legend, main text, or Methods section.

n/a	Confirmed	
☐	☒	The exact sample size (*n*) for each experimental group/condition, given as a discrete number and unit of measurement
☐	☒	A statement on whether measurements were taken from distinct samples or whether the same sample was measured repeatedly
☐	☒	The statistical test(s) used AND whether they are one- or two-sided<br>*Only common tests should be described solely by name; describe more complex techniques in the Methods section.*
☐	☒	A description of all covariates tested
☐	☒	A description of any assumptions or corrections, such as tests of normality and adjustment for multiple comparisons
☐	☒	A full description of the statistical parameters including central tendency (e.g. means) or other basic estimates (e.g. regression coefficient) AND variation (e.g. standard deviation) or associated estimates of uncertainty (e.g. confidence intervals)
☐	☒	For null hypothesis testing, the test statistic (e.g. *F*, *t*, *r*) with confidence intervals, effect sizes, degrees of freedom and *P* value noted<br>*Give P values as exact values whenever suitable.*
☒	☐	For Bayesian analysis, information on the choice of priors and Markov chain Monte Carlo settings
☒	☐	For hierarchical and complex designs, identification of the appropriate level for tests and full reporting of outcomes
☒	☐	Estimates of effect sizes (e.g. Cohen's *d*, Pearson's *r*), indicating how they were calculated

Our web collection on statistics for biologists contains articles on many of the points above.

Software and code

Policy information about availability of computer code

Data collection	Pacific Bioscience SMRTAnalysis pipeline version 9.0.0.92188<br>Flye version 2.8 (https://github.com/fenderglass/Flye)
Data analysis	Genes in Genomes Map (https://github.com/FredHutch/gig-map)<br>Analysis and Visualization platform for microbial 'omics (https://github.com/FredHutch/nf-anvio-pangenome)<br>Partitioned PanGenome Graph of Linked Neighbors (https://github.com/labgem/PPanGGOLiN)<br>kSNP 3.0<br>MEGA X 10.1.8<br>Interactive Tree of Life (iTOL) version 5<br>Operon ConTextualization Across Prokaryotes to Uncover Synteny (OCTAPUS) (github.com/FredHutch/octapus)<br>Prokaryotic Antiviral Defense LOCator (PADLOC) v1.0.0<br>PHAge Search Tool Enhanced Version<br>R packages (prcomp function in stats package, version 3.6.2. PCA function in factoextra package, version 1.0.7)<br>GraphPad Prism 7.0 Software<br>Imaris Microscopy Image Analysis Software<br>Fiji Microscopy Image Analysis Software version 2.1.0/1.53c<br>Metabolon Inc. Analysis Portal<br>MetaboAnalyst

For manuscripts utilizing custom algorithms or software that are central to the research but not yet described in published literature, software must be made available to editors and reviewers. We strongly encourage code deposition in a community repository (e.g. GitHub). See the Nature Portfolio guidelines for submitting code & software for further information.

Data

Policy information about availability of data

All manuscripts must include a data availability statement. This statement should provide the following information, where applicable:

- Accession codes, unique identifiers, or web links for publicly available datasets
- A description of any restrictions on data availability
- For clinical datasets or third party data, please ensure that the statement adheres to our policy

All genomes from this study are available in NCBI under the Bioproject accession number PRJNA549513 and all methylomes are available in REBASE. Raw sequencing data from RNA sequencing experiments are available in the NCBI Sequence Read Archive (SRA) repository under the Bioproject accession number PRJNA937266. Raw sequencing data from 16S rRNA sequencing experiments are available in the NCBI SRA repository under the Bioproject accession number PRJNA1064180.

Human research participants

Policy information about studies involving human research participants and Sex and Gender in Research.

Reporting on sex and gender	For patient tissue specimens included in this study, patient age, gender, or ethnicity were not selection criteria for specimen acquisition.
Population characteristics	This data was not collected.
Recruitment	*Describe how participants were recruited. Outline any potential self-selection bias or other biases that may be present and how these are likely to impact results.*
Ethics oversight	Use of patient tissue specimens was approved by the Fred Hutchinson Cancer Center Institutional Review Board (IRB) under protocols IRB RG1121662, IRB RG1006552, and IRB RG1005305

Note that full information on the approval of the study protocol must also be provided in the manuscript.

Field-specific reporting

Please select the one below that is the best fit for your research. If you are not sure, read the appropriate sections before making your selection.

☒ Life sciences ☐ Behavioural & social sciences ☐ Ecological, evolutionary & environmental sciences

For a reference copy of the document with all sections, see nature.com/documents/nr-reporting-summary-flat.pdf

Life sciences study design

All studies must disclose on these points even when the disclosure is negative.

Sample size	Number of bacterial strains used in this study was determined by available number of genetically unique strains.<br>Sample size for RNA Sequencing (n=3) was based on recommendations from sequencing supplier to allow differential expression analysis.<br>The number of mice used in our murine model studies for pathology was based on a power calculation that determined that if a 40% difference in the mean number of intestinal adenomas between study arms was detected, to obtain an alpha level of 0.02 with 95% power, 8 mice per arm were required.<br>The number of mice used in our murine model studies for metabolomics, was based on a number of intestinal tissues to be analyzed by LC-MS for metabolites based on recommendation from Metabolon, Inc to allow differential analysis between treatment arms (n=4 mice/arm).<br>The number of patient tissue specimens used for microbial analysis was based on available specimens.
Data exclusions	There were no data exclusions.
Replication	All in-vitro functional assays the experiments were conducted at least three times, except for Biolog PM10 Phenotype Microarray Plates which were conducted in duplicate, for data reproducibility. All replicates successfully showed consistent results.
Randomization	Sample randomization into experimental groups is not relevant, as the design of the study aims to quantify features between already established groups.
Blinding	For counting of intestinal adenomas from murine studies, pathologist reviewed tissue sections in a blinded fashion.<br>For all other experiments and analysis, blinding into experimental groups is not relevant, as the design of the study aims to quantify features between already established groups.

Reporting for specific materials, systems and methods

We require information from authors about some types of materials, experimental systems and methods used in many studies. Here, indicate whether each material, system or method listed is relevant to your study. If you are not sure if a list item applies to your research, read the appropriate section before selecting a response.

Materials & experimental systems

n/a	Involved in the study	
☒	☐	Antibodies
☐	☒	Eukaryotic cell lines
☒	☐	Palaeontology and archaeology
☐	☒	Animals and other organisms
☒	☐	Clinical data
☒	☐	Dual use research of concern

Methods

n/a	Involved in the study	
☒	☐	ChIP-seq
☒	☐	Flow cytometry
☒	☐	MRI-based neuroimaging

Eukaryotic cell lines

Policy information about cell lines and Sex and Gender in Research

Cell line source(s)	HCT116 cells were purchased from the American Type Culture Collection (ATCC)
Authentication	This cell line was not authenticated
Mycoplasma contamination	Mycoplasma testing was performed independently by the Research cell bank facility at the Fred Hutch using the MycoProbe Mycoplasma Detection Kit (R&D systems) that can detect the 16S ribosomal RNA of the most common strains of mycoplasma. All cell lines used in this study tested negative for Mycoplasma.
Commonly misidentified lines (See ICLAC register)	No commonly misidentified cell lines were used in the study

Animals and other research organisms

Policy information about studies involving animals; ARRIVE guidelines recommended for reporting animal research, and Sex and Gender in Research

Laboratory animals	Apc Min+/- mice, females, 6-8 weeks old at the start of trial. Mice were housed on a 12- hour light/12-hour dark cycle with controlled temperature (65-75°F (~18-23°C)) and humidity (40-60%).
Wild animals	No wild animals were used in this study.
Reporting on sex	All mice used in this study were female
Field-collected samples	There were no field-collected samples acquired or used in this study.
Ethics oversight	The Fred Hutchinson Cancer Center Animal Care and Use Committee approved all experimental protocols (IACUC PROTO202100004)

Note that full information on the approval of the study protocol must also be provided in the manuscript.
